# Reported long-term effects of COVID-19 patients after hospital discharge in Jordan

**DOI:** 10.1097/MD.0000000000034633

**Published:** 2023-09-22

**Authors:** Reema Karasneh, Sarah Al Sharie, Sayer Al-Azzam, Shoroq M. Altawalbeh, Basheer Khassawneh, Muna Talafha, Mohammad Nusair, Abdel-Hameed Al-Mistarehi, Othman Beni Yonis, Mousa A. Al-Omary, Suad Kabbaha, Mamoon A. Aldeyab

**Affiliations:** a Department of Basic Medical Sciences, Faculty of Medicine, Yarmouk University, Irbid, Jordan; b Faculty of Medicine, Yarmouk University, Irbid, Jordan; c Department of Clinical Pharmacy, Jordan University of Science and Technology, Irbid, Jordan; d Department of Internal Medicine, Faculty of Medicine, Jordan University of Science and Technology, Irbid, Jordan; e Princess Basma Teaching Hospital, Irbid, Jordan; f Department of Pharmacy Practice, Faculty of Pharmacy, Yarmouk University, Irbid, Jordan; g Johns Hopkins University School of Medicine, Baltimore, USA; h Department of Public Health and Family Medicine, Faculty of Medicine, Jordan University of Science and Technology, Irbid, Jordan; i Department of Health Research Methods, Evidence & Impact (HEI), McMaster University, Hamilton, ON, Canada; j Department of Pharmacy, School of Applied Sciences, University of Huddersfield, Huddersfield, UK.

**Keywords:** COVID-19, COVID-19 recovery, long term effects, post COVID-19 syndrome

## Abstract

Coronavirus Disease 2019 (COVID-19) long-term effect is the new challenge facing healthcare providers that should be further assessed. We aim to describe the characteristics and patterns of long-term consequences of COVID-19 among recovered patients. COVID-19 patients baseline data was extracted from hospital records and alive patients filled self-reported symptoms questionnaires. A follow-up chest X-ray (CXR) was then scored based on lung abnormalities and compared with baseline CXR images. Six hundred ninety-four patients were included for the questionnaire and final analysis. Patients who were categorized as critical or severe were more prone to develop at least one symptom than those who were categorized as moderate. The most newly diagnosed comorbidities after discharge were diabetes (40.9%), cardiovascular diseases (18.6%), and hypertension (11.9%). Most patients with prolonged symptoms after discharge had a significant decrease in the quality of life. Small number of CXR showed persistent abnormalities in the middle right, the lower right, and lower left zones with an average overall score during admission 13.8 ± 4.9 and 0.3 ± 1.01 for the follow-up images. Effects of COVID-19 were found to persist even after the end of the infection. This would add on to the disease burden and would foster better management.

## 1. Introduction

The emergence of the novel Severe Acute Respiratory Syndrome Coronavirus 2 (SARS-CoV-2), which is also known as the causative virus of Coronavirus Disease 2019 (COVID-19), was firstly reported in the late 2019 in Wuhan, Hubei, China, and manifested as a cluster of pneumonia cases.^[1]^ It has spread out globally, with fast transmission speed, and developed into a more serious medical concern over time (2). It was announced by the World Health Organization on March 11, 2020 as a global pandemic that requires combined efforts and maximum measures to contain.^[2]^ The spectrum of the severity of symptoms and clinical manifestations in individuals affected by this virus is wide, ranging from an asymptomatic status and reaching to severe levels of illness and sometimes death.^[3–5]^

Until this day, and despite the increased preventive measures and rapid vaccination movement in most of the countries, COVID-19 has been resulting in a massive number of casualties around the world and leading to long-term consequences affecting the quality of life of surviving patients.^[6–8]^ As of September 12, 2022, more than 605 million people have been infected and around 6.49 million have unfortunately died.^[9]^ The SARS-CoV-2 virus enters the human body via the binding of the S glycoprotein spikes of the virus to the angiotensin converting enzyme 2 (ACE2) receptor or a cluster of differentiation 290 L on various human cell surfaces.^[10,11]^ The nasal cavity and alveoli epithelial cells are the main entrance sites for SARS-CoV-2. The kidneys, heart, and intestine may carry ACE2 receptors on its epithelial cell surfaces making these organs susceptible to be affected by the virus alongside the most commonly affected ACE2 receptor rich respiratory system.^[12]^ However, the pathophysiology of pulmonary fibrosis and coagulation in COVID-19 patients is not well understood but it might be explained by the abundance of angiotensin II that is caused by the downregulation of ACE2 enzyme and loss of its activity.^[13]^ Direct viral toxicity, damage to endothelial cells, dysregulation of immune response, and thrombosis-associated inflammation may explain the resultant extrapulmonary manifestation of COVID-19 infection.^[14]^ The number of deaths divided by the number of confirmed cases is defined as the case-fatality rates which can range from 1% to 7% for COVID-19 patients. Based on that, the number of recovered patients from COVID-19 infection is relatively large.^[15,16]^

Focused efforts have studied and described a clear view on the characteristics, pathogenesis, and complications of patients with COVID-19.^[17]^ Previous studies have investigated the long-term effects of this virus and revealed a strong association between the infection with COVID-19 and the development of different symptoms in various body systems.^[18]^ Nevertheless, the long-term health consequences and patterns of this virus remain vague in many areas.^[19,20]^ A better understanding of long-term sequalae of COVID-19 infection will allow health care facilities to provide more efficient multidisciplinary healthcare services navigated to meet patients’ individual needs.^[21]^ Therefore, in this study, we aim to describe the characteristics and patterns of long-term consequences of COVID-19 among recovered patients.

## 2. Methods

### 2.1. Study design and participants

A retrospective cohort study was conducted that included a cohort comprising adult patients with confirmed COVID-19 admitted to King Abdullah University Hospital (KAUH), Jordan between September 2020 and July 2021. After discharge, recovered patients were contacted through phone interview and were asked to participate in the study. Consent was obtained from all participating patients at the beginning of the interview. All participants who were included in this study and had a baseline chest X-ray were invited for X-ray group follow-up. The study used Strengthening the Reporting of Observational studies in Epidemiology cohort reporting guidelines^[22]^ and was approved by the Institutional Review Board of Jordan University of Science and Technology (27/137/2021). Patients who were deceased before the interview, who were unreachable, who were admitted for quarantine reasons at the beginning of the pandemic but had no need for hospitalization in addition to infants and adolescents were all excluded from the study population.

### 2.2. Procedures and outcomes

Baseline clinical data was retrospectively collected from electronic medical records of discharged COVID-19 patients at KAUH and included demographics (age, gender, and smoking status), comorbidities, used treatments and chest X-ray (CXR) images during admission. Extracted data from electronic records were used to avoid any potential of recall bias. Smoking status, new onset comorbidities, and the control of preexisting comorbidities were compared with baseline data. Disease severity was established based on COVID-19 treatment guidelines by the National Institution of Health that categorize patients by severity as mild, moderate, severe, and critical patients.^[23]^ Long-term effects of COVID-19 and symptom duration after infection were categorized into acute (<4 weeks), subacute (4–12 weeks), and chronic (more than 12 weeks).^[24–26]^

Follow up phone interviews were conducted by clinically trained general physicians and patients were asked to complete the survey that included symptoms survey which examined the presence of different symptoms, the starting onset of symptom, duration, and status by the time of the interview, the modified British Medical Research Council dyspnea scale, and the EuroQol five-dimension five-level (EQ-5D-5L) quality of life scale.

The self-reported symptoms survey was built up to measure different aspects of symptoms progression in COVID-19 patients discharged from KAUH. Patients were asked about their demographics (age, gender, current smoking status, marital status, education, employment, nationality), vaccination information, the use of supplement O2 therapy after discharge, the incidence of reinfection or readmission for COVID-19 after discharge, the new diagnosis of any chronic diseases, and whether there was a change in the control of preexisting chronic diseases. In addition, patients were asked to report the occurrence of any new or progressive symptoms before and after the COVID-19 infection. A copy of the self-reported symptoms survey is provided in both English and Arabic (see Table S1, Supplemental Digital Content, which illustrates the self-reported symptoms questionnaire, http://links.lww.com/MD/J608). Patients who responded to have experienced dyspnea as a symptom were asked to complete the modified British Medical Research Council dyspnea scale, which consists of 5 level categories and is used to determine the level of dyspnea based on the physical activity of an individual where increased dyspnea is reflected as a higher level.^[27]^ The EQ-5D-5L is a verified questionnaire established to assess patients’ quality of life based on 5 dimensions (mobility, selfcare, usual activities, pain/discomfort, and anxiety/depression) with each dimension being divided into 5 sublevels ranging from no problems in the specific dimension all the way to extreme problems.^[28]^

A follow-up CXR was conducted, screened and compared with baseline CXR images. Each CXR was divided into an upper, lower, and middle regions both on the right and left for a total of 6 regions. Based on the detected lung abnormalities, a score from 0 to 3 was set for each zone. Score (0) represents no abnormalities. Score (1) was assigned for focal or extensive opacity and septal thickenings with the evidence of extravascular structure. Score (2) represents predominance of interstitial and alveolar infiltrates within the interstitium. Finally, score (3) indicates predominance of interstitial and alveolar infiltrates within the alveolar space. A sum of the scores of the 6 zones with a maximum score of 18 was established reflecting the degree of abnormalities of the full CXR in general. An additional analysis by follow-up period was also conducted for patients with less than 1 year follow-up and more than 1 year follow-up.

### 2.3. Statistical analysis

Descriptive statistics were used for patient characteristics and clinical features of the study sample in total and by admission severity status. Frequency with percentage and arithmetic mean with standard deviation were also used to describe categorical variables and continuous variables, respectively. Continuous variables were assessed for normality using histograms, Quantile-Quantile plots in addition to Kolmogorov–Smirnov and the Shapiro–Wilk tests. Patient characteristics and clinical features were compared across admission severity groups using chi-square test and the analysis of variance test as appropriate.

The incidence of post COVID-19 symptoms was compared across admission severity groups using chi-square test. Logistic regression models were conducted to calculate the odds ratios for these symptoms in order to assess possible associations between them and admission severity. Multivariable logistic regression models were conducted to evaluate the impact of admission severity on the occurrence of post COVID-19 symptoms adjusting for potential confounders selected using backward stepwise process with *P* < .2 to stay. The average X-ray total score was compared during and post admissions using paired t test and lung zone abnormalities were compared using McNemar test.

Utility score (EQ-5D-5L utility) ranging from 0 to 1 was calculated for each patient at time of interview. EQ-5D was scored using United Kingdom general population scoring algorithm (i.e., EQ-5D-5L Crosswalk Index Value Calculator). The average EQ-5-DL score in patients with the symptom was compared with the average score in patients without the symptom. The two-sample t-test was used to evaluate associations between utility scores and having different post COVID-19 symptoms.

All data analyses were conducted using Stata version 17 software (StataCorp. 2021. Stata: Release 17. Statistical Software. College Station, TX: StataCorp LLC.). The statistical significance was set at a 2-sided *P* < .05.

## 3. Results

**Figure 1.**
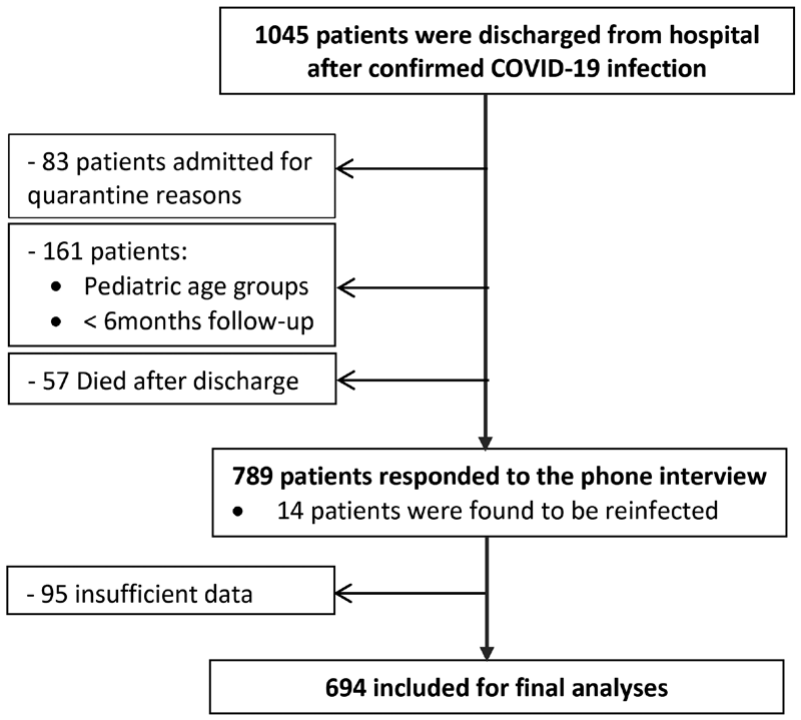
COVID-19 patients’ recruitment process.

A total of 1045 patients were discharged from KAUH, Irbid, Jordan during the study period. Patients’ recruitment process is shown in Figure 1. A total of 694 patients were included for the questionnaire and final analysis. Clinical characteristics and demographics of patients are demonstrated in Table 1. The mean age of the study patients was 58.4 years with around 60% of patients were males. The majority of patients were married (82.1%) and of Jordanian nationality (96.0%). The number of patients who were smokers decreased from 89 at the time of admission to 74 patients at the time of the interview. The majority of patients received their vaccination after their discharge (74. 8%) and 28 of them were vaccinated before admission (4.0%); the rest were not vaccinated at the time of the interview. Prophylactic low molecular weight heparin and glucocorticoids were used with the vast majority of admitted patients (97.4%, 96.0% respectively). The most common comorbidity among the study population at baseline was hypertension (53.5%) followed by Diabetes Mellitus (DM) (46.0%), and cardiovascular diseases (18.9%). The mean duration of stay at the hospital was 9.6 days and the median of the duration of the time from discharge until follow-up was 345.2 days (11.3 months). Higher percentages of immunocompromised patients were of severe cases (66.7%) compared to critical (26.7%) and moderate COVID-19 cases (6.7%).

**Table 1:** General characteristics of the study population.

	Total (%)	Severity of illness (%)			*P*-value
		Moderate	Severe	Critical	
Age, years	58.4 ± 14.3	58.0 ± 14.8	58.6 ± 14.9	58.5 ± 13.8	.989
Gender					.112
Male	413/694 (59.5)	67/413 (16.2)	141/413 (34.1)	205/413 (49.6)	
Female	281/694 (40.5)	55/281 (19.6)	109/281 (38.8)	117/281 (41.6)	
Marital status					.188
Single	29/694 (4.2)	4/29 (13.8)	11/29 (37.9)	14/29 (48.3)	
Married	570/694 (82.1)	95/507 (16.7)	202/570 (35.4)	273/570 (47.9)	
Widowed	93/694 (13.4)	23/93 (24.7)	35/93 (37.6)	35/93 (37.6)	
Divorced	2/694 (0.3)	0/2 (0.0)	2/2 (100.0)	0/2 (0.0)	
Educational level					.524
College or higher	334/694 (48.1)	63/334 (18.9)	114 (34.1)	334 (47.0)	
High school or lower	360/694 (51.9)	59/360 (16.4)	136/360 (37.8)	165/360 (45.8)	
Employment					.543
Employed	215/694 (31.0)	35/215 (16.3)	70/215 (32.6)	110/215 (51.2)	
Unemployed	291/694 (41.9)	53/291 (18.2)	107/291 (36.8)	131/291 (45.0)	
Retired	188/694 (27.1)	34/188 (18.1)	73/188 (38.8)	81/188 (43.1)	
Nationality					.322
Jordanian	666/694 (96.0)	120/666 (18.0)	238/666 (35.7)	308/666 (46.3)	
Non-Jordanian	28/694 (4.0)	2/28 (7.1)	12/28 (42.9)	14/28 (50.0)	
Smoking status at time of interview					.052
Current smoker	74/694 (10.7)	10/74 (13.5)	19/74 (25.7)	45/74 (60.8)	
Ex-smoker	153/694 (22.1)	25/153 (16.3)	65/153 (42.5)	63/153 (41.2)	
Not a smoker	467/694 (67.3)	87/467 (18.6)	166/467 (35.6)	214/467 (45.8)	
Vaccination info					.308
Vaccinated	547/694 (78.8)	94/547 (17.2)	191/547 (34.9)	262/547 (47.9)	
Not vaccinated	147/694 (21.2)	28/147 (19.1)	59/147 (40.1)	60/147 (40.8)	
Vaccination time					.322
Before admission	28/694 (4.0)	2/28 (7.1)	10/28 (35.7)	16/28 (57.1)	
After admission	519/694 (74.8)	92/519 (17.7)	181/519 (34.9)	246/519 (47.4)	
Baseline comorbidities					
Hypertension	371/694 (53.5)	65/371 (17.5)	139/371 (37.5)	167/371 (45.0)	.673
Diabetes Mellitus	319/694 (46.0)	52/319 (16.3)	125/319 (39.2)	142/319 (44.5)	.267
Dyslipidemia	32/694 (4.6)	4/32 (12.5)	14/32 (43.8)	14/32 (43.8)	.577
Cardiovascular disease	131/694 (18.9)	25/131 (19.1)	46/131 (35.1)	60/131 (45.8)	.879
Cerebrovascular disease	30/694 (4.3)	6/30 (20.0)	15/30 (50.0)	9/30 (30.0)	.066
Chronic kidney disease	37/694 (5.3)	11/37 (29.7)	14/37 (37.8)	12/37 (32.4)	.080
Chronic liver disease	1/694 (0.1)	1/1 (100.0)	0/1 (0.0)	0/1 (0.0)	.352
Thromboembolic disorders	5/694 (0.7)	0/5 (0.0)	3/5 (60.0)	2/5 (40.0)	.420
Asthma	20/694 (2.9)	1/20 (5.0)	6/20 (30.0)	13/20 (65.0)	.166
Chronic obstructive pulmonary disease	6/694 (0.9)	3/6 (50.0)	2/6 (33.3)	1/6 (16.7)	.091
Obstructive sleep apnea	5/694 (0.7)	1/5 (20.0)	4/5 (80.0)	0/5 (0.0)	.080
Malignant tumor	29/694 (4.2)	5/29 (17.2)	14/29 (48.3)	10/29 (34.5)	.335
Immunocompromised (immunosuppressive therapy/ active chemotherapy)	15/694 (2.2)	1/15 (6.7)	10/15 (66.7)	4/15 (26.7)	**.042**
Rheumatoid arthritis	6/694 (0.9)	1/6 (16.7)	2/6 (33.3)	3/6 (50.0)	.984
Hypothyroidism	30/694 (4.3)	9/30 (30.0)	9/30 (30.0)	12/30 (40.0)	.188
Gout	30/694 (4.3)	5/30 (16.7)	9/30 (30.0)	16 (53.3)	.720
Treatment received during hospital stay					
Glucocorticoids	666/694 (96.0)	102/666 (15.3)	244/666 (36.6)	320/666 (48.1)	**<.001**
Azithromycin	57/694 (8.2)	15/57 (26.3)	16/57 (28.1)	26/57 (45.6)	.150
Prophylactic low molecular weight heparin	676/694 (97.4)	109/676 (16.1)	246/676 (36.4)	321/676 (47.5)	**<.001**
Anti-platelets	289/694 (41.6)	53/289 (18.3)	103/289 (35.6)	133/289 (46.0)	.866
Antihypertensives	404/694 (58.2)	68/404 (16.8)	141/404 (34.9)	195/404 (48.3)	.244
Diuretics	193/694 (27.8)	38/193 (19.7)	62/193 (32.1)	93/193 (48.2)	.558
Statins	216/694 (31.1)	38/216 (17.6)	70/216 (32.4)	108/216 (50.0)	.365
Length of hospital stay, days	9.6 ± 8.3	8.1 ± 8.0	7.9 ± 6.6	11.4 ± 9.2	**<.001**
Time from discharge to follow-up, days	345.2 ± 70.3	377.3 ± 57.4	357.0 ± 66.2	323.8 ± 71.2	**<.001**

Bold values indicate statistical significance of the results. They are statistically significant *P* < .05.

**Figure 2.**
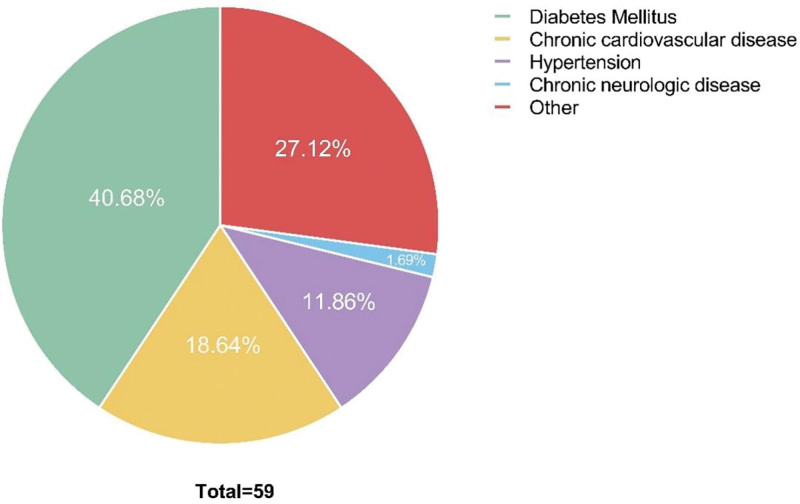
Newly diagnosed comorbidities after discharge.

One third of patients (35.0%) required O2 supplement after discharge and it lasted for a mean of 20 days. Fifty-nine patients (8.5%) were found to be newly diagnosed with at least 1 comorbidity after their discharge. As shown in Figure 2, DM was the most newly encountered comorbidity after discharge (40.9%), followed by chronic cardiovascular diseases, and hypertension (18.6%, and 11.9% respectively). Of the patients who used to have DM before admission, 240 patients (75.2%) have not noticed any change in their control of the disease, 66 patients (20.7%) had a worse control than before admission, and 13 (4.1%) reported a better control of their conditions. When investigating the control over hypertensive patients, 314 patients (84.6%) have not noticed any change in their control of the disease, 46 patients (12.4%) had a worse control than before admission, and only 11 patients (3.0%) reported a better control of their conditions.

Table 2 shows that 649 of the discharged patients (93.5%) reported experiencing at least 1 symptom. The most common experienced symptom by patients was extreme fatigue (73.4%) followed by dyspnea (62.1%), cough (51.6%), and changes in mood (Anxiety/ depression) (43.1%). Among these patients whose their symptoms started after COVID-19 admission, critical cases had lower odds of losing their taste and smell compared to moderate cases (OR = 0.57, 95% CI 0.36–0.9, OR = 0.59, 95% CI 0.37–0.93, respectively) and likewise severe cases compared to moderate cases (OR = 0.45, 95% CI 0.29–0.70, OR = 0.44, 95% CI 0.28–0.69, respectively). Sputum production, high grade fever, and abdominal pain were significantly different by severity status. In the additional analysis conducted by follow-up period (see Tables S2 and S3, Supplemental Digital Content, which illustrate symptoms for <1 year follow-up duration and symptoms for more than 1 year follow-up duration, respectively, http://links.lww.com/MD/J609, http://links.lww.com/MD/J610), interviews conducted less than 1 year post discharge, higher severity was significantly protective (decrease likelihoods) for only sputum production and abdominal pain. On the other hand, for interviews conducted later than 1 year post discharge, higher severity was positively (increase likelihoods) for cough, dyspnea, sore throat, and palpitation.

**Table 2:** Symptoms, exercise capacity, and health-related quality of life at follow-up according to severity scale.

		Severity of illness (%)			OR (95% CI)	
Symptoms	Total (n = 694)	Moderate (n = 122)	Severe (n = 250)	Critical (n = 322)	Severe versus moderate	Critical versus moderate
Any of the following symptoms	649/694 (93.5)	113/122 (92.6)	232/250 (92.8)	304/322 (94.4)	1.03 (0.45–2.36)	1.35 (0.59–3.08)
Extreme fatigue	503/685 (73.4)	91/120 (75.8)	187/247 (75.7)	225/318 (70.8)	0.99 (0.6–1.65)	0.77 (0.48–1.25)
Cough	353/684 (51.6)	58/121 (47.9)	142/248 (57.3)	153/315 (48.6)	1.46 (0.94–2.25)	1.03 (0.67–1.56)
Hemoptysis	24/694 (3.5)	5/122 (4.1)	7/250 (2.8)	12/322 (3.7)	0.67 (0.21–2.17)	0.91 (0.31–2.63)
Sputum production	157/686 (22.9)	35/122 (28.7)	60/248 (24.2)	62/316 (19.6)	0.79 (0.49–1.29)	**0.61 (0.38–0.98**)
Low grade fever	15/694 (2.2)	2/122 (1.6)	7/250 (2.8)	6/322 (1.9)	1.73 (0.35–8.45)	1.14 (0.23–5.72)
High grade fever	183/694 (26.4)	37/122 (30.3)	79/250 (31.6)	67/322 (20.8)	1.06 (0.66–1.70)	**0.60 (0.38–0.97**)
Chills	216/694 (31.1)	49/122 (40.2)	88/250 (35.2)	79/322 (24.5)	0.81 (0.52–1.26)	**0.48 (0.31–0.76**)
Nasal congestion	120/690 (17.4)	25/122 (20.5)	48/249 (19.3)	47/319 (14.7)	0.93 (0.54–1.59)	0.67 (0.39–1.15)
Nosebleed	20/692 (2.9)	6/121 (5)	9/250 (3.6)	5/321 (1.6)	0.72 (0.25–2.06)	0.30 (0.09–1.01)
Dyspnea	423/681 (62.1)	70/120 (58.3)	149/245 (60.8)	204/316 (64.6)	1.11 (0.71–1.73)	1.30 (0.85–2.00)
Sore Throat	130/688 (18.9)	21/122 (17.2)	40/248 (16.1)	69/318 (21.7)	0.92 (0.52–1.65)	1.33 (0.78–2.29)
Chest pain	227/684 (33.2)	47/120 (39.2)	78/246 (31.7)	102/318 (32.1)	0.72 (0.46–1.14)	0.73 (0.47–1.13)
Palpitation	141/657 (21.5)	22/117 (18.8)	50/241 (20.8)	69/299 (23.1)	1.13 (0.65–1.98)	1.30 (0.78–2.21)
Changes mood Anxiety/Depression	281/652 (43.1)	53/115 (46.1)	103/235 (43.8)	125/302 (41.4)	0.91 (0.58–1.43)	0.83 (0.54–1.27)
Headache	182/675 (27)	36/116 (31)	72/245 (29.4)	74/314 (23.6)	0.92 (0.57–1.49)	0.69 (0.43–1.1)
Seizures	1/694 (0.1)	0/122 (0)	1/250 (0.4)	0 (0)	N/A	N/A
Loss of taste	198/694 (28.5)	50/112 (41)	71/250 (28.4)	77/322 (23.9)	**0.57 (0.36–0.9**)	**0.45 (0.29–0.7**)
Loss of smell	199/691 (28)	50/121 (41.3)	73/249 (29.3)	76/321 (23.7)	**0.59 (0.37–0.93**)	**0.44 (0.28–0.69**)
Diarrhea	123/689 (17.9)	23/122 (18.9)	53/247 (21.5)	47/320 (14.7)	1.18 (0.68–2.03)	0.74 (0.43–1.28)
Vomiting	51/691 (7.4)	12/121 (9.9)	21/248 (8.5)	18/322 (5.6)	0.84 (0.4–1.77)	0.54 (0.25–1.15)
Abdominal pain	75/690 (10.9)	22/120 (18.3)	27/248 (10.9)	26/322 (8.1)	0.54 (0.3–1.00)	**0.39 (0.21–0.72**)
Myalgia	333/585 (56.9)	57/102 (55.9)	127/211 (60.2)	149/272 (54.8)	1.19 (0.74–1.93)	0.96 (0.6–1.51)
Skin rash	43/682 (6.3)	7/115 (6.1)	15/250 (6)	21/317 (6.7)	0.98 (0.39–2.5)	1.09 (0.45–2.65)
Conjunctivitis	21/691 (3)	5/120 (4.2)	9/250 (3.6)	7/321 (2.2)	0.86 (0.28–2.62)	0.51 (0.16–1.65)
mMRC score						
≥1	173/328 (52.7)	29/57 (50.9)	65/117 (55.6)	79/154 (51.3)	1.21 (0.64–2.28)	1.02 (0.55–1.87)

Bold values indicate statistical significance of the results. They are statistically significant *P* < .05.

Table 3 shows the start point, duration and current status of the symptoms. In almost all patients, symptoms that were experienced during admission were resolved after discharge except for skin rash that had higher percentages (49.1%) compared to before admission (21.8%) and during the admission (29.0%) for COVID-19 infection. Patients who stated that they have experienced some symptoms before the onset of COVID-19 were excluded when considering the duration and the current status of each symptom as the aim of this study was to investigate the symptoms that began to appear after the COVID-19 infection. Most patients who complained from different symptoms had their symptoms resolved in the first 4 weeks after infection, while some patients had their symptoms for a duration of 4–12 weeks, and a lesser number in general had symptoms that were persistent for more than 12 weeks. Change in mood was the only symptom where the number of patients with a symptom persisting for more than 12 weeks (45.6%) and was higher than the other 2 duration categories (44.5% for <4 weeks, and 10.0% for 4–12 weeks). Moreover, by the time of phone interview, most of the symptoms had completely resolved, some of them had a better status and a very few numbers had their symptoms to be the same as or worse than the time of admission.

**Table 3:** Symptoms with durations.

	Number/yes (%)	Start period (%)			Duration/symptoms (%)			Current status (%)			
		Before admission	During admission	After discharge	< 4 Weeks	4–12 Weeks	>12 Weeks	Better than before	Completely resolved	Same as before	Worse than before
Generalized fatigue	512/694 (73.8)	9/512 (1.8)	467/512 (91.2)	36/512 (7.0)	205/503 (40.7)	179/503 (35.6)	119/503 (23.6)	105/503 (20.9)	371/503 (73.8)	15/503 (3.0)	12/503 (2.4)
Cough	363/694 (52.3)	10/363 (2.8)	320/363 (88.2)	33/363 (9.1)	197/353 (55.8)	98/353 (27.8)	58/353 (16.4)	38/353 (10.8)	294/353 (83.3)	10/353 (2.8)	11/353 (3.1)
Hemoptysis	24/694 (3.5)	0/24 (0.0)	22/24 (91.7)	2/24 (8.3)	17/24 (70.8)	5/24 (20.8)	2/24 (8.3)	1/24 (4.2)	23/24 (95.8)	0/24 (0.0)	0/24 (0.0)
Sputum production	165/694 (23.8)	8/165 (4.9)	135/165 (81.8)	22/165 (13.3)	84/157 (53.5)	34/157 (21.7)	39/157 (24.8)	30/157 (19.1)	115/157 (73.3)	10/157 (6.4)	2/157 (1.3)
Low grade fever	15/694 (2.2)	0/15 (0.0)	11/15 (73.3)	4/15 (26.7)	11/15 (73.3)	1/15 (6.7)	3/15 (20.0)	1/15 (6.7)	12/15 (80.0)	1/15 (6.7)	1/15 (6.7)
High grade fever	183/694 (26.4)	0/183 (0.0)	183/183 (99.5)	1/183 (0.6)	180/183 (98.4)	3/183 (1.6)	0/183 (0.0)	0/183 (0.0)	183/183 (100.0)	0/183 (0.0)	0/183 (0.0)
Chills	216/694 (31.1)	0/216 (0.0)	200/216 (92.6)	16/216 (7.4)	184/216 (85.2)	15/216 (6.9)	17/216 (7.9)	12/216 (5.7)	198/216 (91.7)	2/216 (0.9)	4/216 (1.9)
Nasal congestion/runny nose	124/694 (17.9)	4/124 (3.2)	99/124 (79.8)	21/124 (16.9)	81/120 (67.5)	11/120 (9.2)	28/120 (23.3)	17/120 (14.2)	88/120 (73.3)	13/120 (10.8)	2/120 (1.7)
Nosebleed	22/694 (3.2)	2/22 (9.1)	15/22 (68.2)	5/22 (22.7)	16/20 (80.0)	2/20 (10.0)	2/20 (10.0)	1/20 (5.0)	18/20 (90.0)	0/20 (0.0)	1/20 (5.0)
Dyspnea	436/694 (62.8)	13/436 (3)	405/436 (93)	18/436 (4.1)	167/423 (39.5)	108/423 (25.5)	148/423 (35.0)	139/423 (32.9)	250/423 (59.1)	22/423 (5.0)	12/423 (2.8)
Sore throat	136/694 (19.6)	6/136 (4.4)	105/136 (77.2)	25/136 (18.4)	91/130 (70.0)	16/130 (12.3)	23/130 (18.0)	16/130 (12.3)	103/130 (79.2)	9/130 (7.0)	2/130 (1.5)
Chest pain	237/694 (34.2)	10/237 (4.2)	196/237 (82.7)	31/237 (13.1)	119/227 (52.4)	46/227 (20.3)	62/227 (27.3)	48/227 (21.2)	160/227 (70.5)	15/227 (6.6)	4/227 (1.8)
Palpitation	178/694 (25.7)	37/178 (20.8)	118/178 (66.3)	23/178 (12.9)	77/141 (54.6)	18/141 (12.8)	46/141 (32.6)	36/141 (25.5)	87/141 (61.7)	10/141 (7)	8/141 (5.7)
Changes in mood	323/694 (46.5)	42/323 (13.0)	216/323 (66.9)	65/323 (20.1)	125/281 (44.5)	28/281 (10)	128/281 (45.5)	88/281 (31.3)	135/281 (48.0)	35/281 (12.5)	23/281 (8.2)
Headache	201/694 (29)	19/201 (9.5)	155/201 (77.1)	27/201 (13.4)	123/182 (67.6)	13/182 (7.1)	46/182 (25.3)	32/182 (17.6)	131/ 182 (72.0)	12/182 (6.6)	7/182 (3.9)
Seizures	1/694 (0.14)	0/1 (0.0)	0/1 (0.0)	1/1 (100.0)	0/1 (0.0)	0/1 (0.0)	1/1 (100.0)	0/1 (0.0)	0/1 (0.0)	1/1 (100.0)	0/1 (0.0)
Loss/taste	198/694 (28.5)	0/198 (0.0)	193/198 (97.5)	5/198 (2.5)	125/198 (63.1)	48/198 (24.2)	25/198 (12.6)	14/198 (7.1)	174/198 (87.9)	9/198 (4.6)	1/198 (0.51)
Loss/smell	202/694 (29.1)	3/202 (1.5)	196/202 (97.0)	3/202 (1.5)	124/199 (62.3)	44/199 (22.1)	31/199 (15.6)	17/199 (8.5)	162/199 (81.4)	16/199 (8.0)	4/199 (2.0)
Diarrhea	128/694 (18.4)	5/128 (3.9)	110/128 (85.9)	13/128 (10.2)	105/123 (85.4)	7/123 (5.7)	11/123 (8.9)	2/123 (1.6)	116/199 (94.31)	4/123 (3.3)	1/123 (0.8)
Vomiting	54/694 (7.8)	3/54 (5.6)	47/54 (87.04)	4/54 (7.4)	47/51 (92.2)	1/51 (2.0)	3/51 (5.9)	0/51 (0.0)	49/51 (96.1)	2/51 (3.9)	0/51 (0.0)
Abdominal pain	79/964 (11.4)	4/79 (5.1)	61/79 (77.2)	14/79 (17.7)	58/75 (77.3)	3/75 (4.0)	14/75 (18.7)	3/75 (4.0)	63/75 (84.0)	6/75 (8.0)	3/75 (4.0)
Myalgia/arthralgia	442/694 (63.7)	109/442 (24.7)	288/442 (65.2)	45/442 (10.2)	145/333 (43.6)	72/333 (21.6)	116/333 (34.8)	78/333 (23.4)	207/333 (62.2)	32/333 (9.6)	16/333 (4.8)
Skin rash	55/694 (7.9)	12/55 (21.8)	16/55 (29.0)	27/55 (49.1)	25/43 (58.1)	8/43 (18.6)	10/43 (23.3)	6/43 (14.0)	31/43 (72.1)	5/43 (11.6)	1/43 (2.3)
Conjunctivitis	24/694 (3.5)	3/24 (12.5)	14/24 (58.3)	7/24 (29.2)	12/21 (57.1)	0/21 (0.0)	9/21 (42.9)	5/21 (23.8)	11/21 (52.4)	2/21 (9.5)	3/21 (14.3)

The average score of the EQ-5-DL in patients with symptoms compared to those without the symptoms are shown in Table 4. General symptoms (generalized fatigue, high grade fever, chills, changes in mood, headache, myalgia/arthralgia), respiratory symptoms (sore throat, nasal congestion/runny nose, cough, dyspnea), cardiac symptoms (chest pain, palpitation), gastrointestinal symptoms (diarrhea, abdominal pain), and conjunctivitis were significantly associated with decreased quality of life in affected patients. Only 1 patient has complained from seizures in the study sample and therefor was not included for further analysis.

**Table 4:** EQ-5D-L utility average comparing the presence with the absence of each symptom.

	With the symptom	Without the symptom	*P*-value
Generalized fatigue	0.74 ± 0.27	0.83 ± 0.24	**<.001**
Cough	0.74 ± 0.72	0.79 ± 0.27	.019
Hemoptysis	0.76 ± 0.23	0.78 ± 0.27	.856
Sputum production	0.74 ± 0.26	0.77 ± 0.27	.196
Low grade fever	0.72 ± 0.33	0.77 ± 0.26	.511
High grade fever	0.72 ± 0.27	0.78 ± 0.26	**.009**
Chills	0.72 ± 0.27	0.79 ± 0.26	**.001**
Nasal congestion/runny nose	0.68 ± 0.3	0.79 ± 0.25	**<.001**
Nosebleed	0.8 ± 0.05	0.77 ± 0.27	.559
Dyspnea	0.73 ± 0.27	0.83 ± 0.25	**<.001**
Sore throat	0.72 ± 0.26	0.78 ± 0.27	**.041**
Chest pain	0.71 ± 0.25	0.79 ± 0.27	**<.001**
Palpitation	0.69 ± 0.28	0.79 ± 0.26	**<.001**
Changes in mood	0.68 ± 0.3	0.84 ± 0.21	**<.001**
Headache	0.71 ± 0.28	0.79 ± 0.26	**<.001**
Seizures	0.028	0.77 ± 0.26	N/A
Loss of taste	0.74 ± 0.25	0.78 ± 0.27	.090
Loss of smell	0.74 ± 0.26	0.78 ± 0.27	.060
Diarrhea	0.72 ± 0.26	0.78 ± 0.27	**.036**
Vomiting	0.71 ± 0.30	0.77 ± 0.26	.120
Abdominal pain	0.68 ± 0.28	0.78 ± 0.26	**.002**
Myalgia/arthralgia	0.73 ± 0.26	0.84 ± 0.26	**<.001**
Skin rash	0.74 ± 0.32	0.77 ± 0.26	.528
Conjunctivitis	0.61 ± 0.27	0.77 ± 0.26	**.003**

Bold values indicate statistical significance of the results. They are statistically significant *P* < .05.

Follow up chest X-ray was conducted for 100 patients, 4 of which were excluded as they did not meet the inclusion criteria. Frequencies and percentage of chest X-rays scores during admissions and after follow-up are shown in Table 5. During the admission, the majority of included patients were found to have developed abnormalities in the different 6 zones of the CXR. After screening, it was found that the score of the CXRs increased in most of the patients whenever a lower zone in each of the right and left sides of the lungs is involved. After the follow up, CXRs of the followed patients were found to tremendously improve, as most of the zones have scored 0, and only minor damage to the lower zones have persisted (Table 5).

**Table 5:** Frequency and percentage of chest X-rays scores during admissions compared to follow-up.

	Admission chest X-ray score	Follow-up chest X-ray score
Upper right zone		
0	10 (10.4%)	96 (100%)
1	29 (30.2%)	0 (0%)
2	0 (0%)	0 (0%)
3	57 (59.4%)	0 (0%)
Upper left zone		
0	14 (14.6%)	96 (100%)
1	31 (32.3%)	0 (0%)
2	0 (0%)	0 (0%)
3	51 (53.1%)	0 (0%)
Middle right zone		
0	6 (6.3%)	96 (100%)
1	23 (24%)	0 (0%)
2	5 (5.2%)	0 (0%)
3	62 (64.6%)	0 (0%)
Middle left zone		
0	7 (7.29%)	94 (97.92%)
1	19 (19.79%)	2 (2.08%)
2	3 (3.13%)	0 (0%)
3	67 (69.79%)	0 (0%)
Lower right zone		
0	3 (3.1%)	86 (89.6%)
1	18 (18.8%)	9 (9.4%)
2	4 (4.2%)	0 (0%)
3	71 (74%)	1 (1%)
Lower left zone		
0	3 (3.1%)	87 (90.6%)
1	8 (8.3%)	6 (63%)
2	1 (1%)	0 (0%)
3	84 (87.5%)	3 (3.1%)

The comparison between CXRs during admission and after discharge showed that the vast majority of patients have had a completely normal CXR at the time of follow-up. A small number of patients showed persistent abnormalities in the middle right, the lower right, and lower left zones. The average overall score during admission was 13.8 ± 4.9 whereas it was only 0.3 ± 1.01 in the follow-up images. (Table 6).

**Table 6:** Number of patients with abnormal chest X-rays findings during admission compared to follow-up.

	During admission (%)	Post discharge (%)	*P*-value
Upper right zone	86 (89.6)	0 (0.0)	**<.001**
Upper left zone	82 (85.4)	0 (0.0)	**<.001**
Middle right zone	90 (93.8)	0 (0.0)	**<.001**
Middle left zone	89 (92.7)	2 (2.1)	**<.001**
Lower right zone	93 (96.9)	10 (10.4)	**<.001**
Lower left zone	93 (96.9)	9 (9.4)	**<.001**
Average score	13.8 ± 4.9	0.3 ± 1.01	**<.001**

Bold values indicate statistical significance of the results. They are statistically significant *P* < .05.

## 4. Discussion

To the best of our knowledge, few studies in the region have investigated the long-term sequalae of COVID-19 in patients required hospital admissions. Our study explores the relationship between disease severity during admission and the likelihood of developing post-COVID-19 symptoms. Furthermore, the impact of post-COVID-19 symptoms on patient quality of life after discharge was also evaluated. In this study, one third of Covid-19 patients reported requiring O2 supplement which lasted for a mean of 20 days and approximately 94% of them reported the development of at least 1 symptom after discharge with extreme fatigue, dyspnea, cough, and changes in mood being the most to be encountered by patients. Patients who developed symptoms after being discharged reported a notable decline in their quality of life in comparison to those who did not experience post-COVID-19 symptoms. On less than 1 year follow-up, patients who had a severe illness during admission were less likely to experience post-COVID-19 symptoms such as sputum production and abdominal pain when compared to those who had a moderate illness. Finally, most patients had very high CXR scores during admission in comparison to clear CXRs after discharge regardless of disease severity.

For some patients, COVID-19 can result in different symptoms that may last for various durations of time after the resilience of the infection itself.^[29]^ The consequences and long-term effects of COVID-19 have not been identified sufficiently and the spectrum of long-term clinical manifestations can range from asymptomatic up to highly fatal end organ dysfunction.^[30]^ In our study, the most encountered symptoms were noticed to be generalized fatigue followed by myalgia and arthralgia.

According to the National Health Service (NHS) in the United Kingdom (UK), post-COVID syndrome is defined as enigmatic everlasting signs or symptoms that rise during or after COVID-19 infections and persisting for more than 12 weeks.^[31]^ Symptoms of post-COVID syndrome are commonly overlapping with approximately 80% of patients experiencing more than 2 and up to 10 symptoms.^[32]^ The prevalence of this syndrome is wide and ranges from 13.3% to 96.0% with the majority of studies reporting higher rates.^[33–39]^ This is similar to our findings in which the presence of any post COVID-19 symptom was found in 93.5% of the participants.

In a multi-centered observational study that investigated the outcomes of hospitalized patients at 60 days post discharge from 38 hospitals showed that 6.7% of the patients who were discharged had died and 15.1% were hospitalized at least once more after their discharge. Of the patients who completed the survey, 32.6% had persistent symptoms and 18.9% had complained of new or worsened symptoms.^[40]^ Lower mortality rate was observed in our study with 23.6 % had persistent symptoms for more than 12 weeks after admission. In Carfì et al study of 143 discharged patients, only 12.6% have reported the absence of any symptoms after COVID-19 infection and 87.4% have reported the persistence of symptoms after a mean of 60 days from the onset of symptoms. Furthermore, 44.1% of patients reported a decrease in the quality of life.^[33]^ This is consistent with our findings in which patients who experienced symptoms after discharge complained of a significant decrease in the quality of life when compared to those who did not experience post COVID-19 symptoms.

Similar to our findings, several studies that investigated the long-term effects and symptoms of COVID-19 found that fatigue, dyspnea, arthralgia and myalgia were the most common symptoms after discharge.^[39,41–43]^ Respiratory and functional impairment were also reported among patients with COVID-19 4 months after hospital discharge in a prospective cohort study that included 238 patients.^[44]^ It has been reported that cough can persist for weeks or even months after COVID-19 infection, however, the presence of cough after SARS-CoV-2 infection is much less common than dyspnea and has a prevalence ranging from 2% to 42% of patients.^[45]^ This is consistent with our findings in which dyspnea was found to be more commonly persistent in patients discharged after COVID-19 when compared to cough. Olfactory function was assessed in a multicenter cohort study that investigated the long-term consequences of COVID-19 and included 1363 patients. Olfaction was found to be recovered after 2 and 6 months among 75% and 95% of patient respectively.^[46]^ This is similar to our findings in which most patients who complained from olfactory dysfunction have completely retained their ability to smell.

In a large prospective cohort study, more severe long-term consequences of COVID-19 including impaired pulmonary diffusion capacities and abnormal chest imaging manifestations were observed among patients with increased severity during admission.^[18]^ Similarly, Prnjavoracet et al reported that the degree of persistence of chest x-ray abnormal findings were associated with the severity of the disease during admission.^[47]^ Furthermore, in a retrospective cohort study by Jovanoski et al an increased risk of new clinical conditions was observed in patients with severe COVID-19.^[48]^ However, reassuring findings were observed by Townsend et al that concluded that outcomes and effects of COVID-19 on the long run are not associated with the severity of disease.^[49]^ This is consistent with our findings in which significant lower odds were observed for several symptoms of COVID-19 in severe cases compared to moderate cases. Fogante and colleagues evaluated the effects of COVID-19 on the long run from a radiological point of view. Patients who had completely resolved CXR at midterm follow-up were found to have lower CXR scores at admission as well as at discharge when compared to those who have some abnormalities on CXR at midterm follow-up.^[50]^ Most patients in our study had very high scores during admission in comparison to clear CXRs after discharge regardless of disease severity.

Our study had large sample size with extended follow up duration compared to published literature, and a comprehensive self-reported symptoms questionnaire obtained by well-trained general physicians. These well-established baseline characteristics are necessary for future prospective cohort studies that are required to determine whether these long-term effects either complicate previous diseases or are a continuation of COVID-19.^[32]^ It also fills a critical knowledge gap in the current literature by focusing specifically on the long-term effects of COVID-19 patients after hospital discharge in the region.^[51]^ However, a number of patients were unreachable and were lost to follow-up thus they were not included in this cohort and those who were invited to the chest X-rays and did not participate in the study may have produced a source of selection bias. Also, follow-up chest computed tomography scan is usually considered more sensitive and specific than chest x-ray in evaluating the pulmonary radiological manifestations but due to lack of a baseline computed tomography images in most patient, chest X-ray was preferred to be used.

## 5. Conclusion

Individuals discharged from the hospital after acute COVID-19 infection will mostly experience at least 1 symptom after discharge with fatigue and cough being usually bothersome symptoms and mostly resolve within the first 4 weeks after discharge. Persistent or new symptoms post COVID-19 infection were found to have a profound effect on patient’s quality of life.

## Author contributions

**Conceptualization:** Reema Karasneh, Sarah Al Sharie, Sayer Al-Azzam, Shoroq M. Altawalbeh, Basheer Khassawneh, Muna Talafha, Mohammad Nusair, Abdel-Hameed Al-Mistarehi, Othman Beni Yonis, Mousa A. Al-Omary, Suad Kabbaha, Mamoon A. Aldeyab.

**Data curation:** Reema Karasneh, Sarah Al Sharie, Sayer Al-Azzam, Basheer Khassawneh, Muna Talafha, Mohammad Nusair, Abdel-Hameed Al-Mistarehi, Othman Beni Yonis, Suad Kabbaha, Mamoon A. Aldeyab.

**Formal analysis:** Reema Karasneh, Sayer Al-Azzam, Shoroq M. Altawalbeh, Suad Kabbaha, Mamoon A. Aldeyab.

**Funding acquisition:** Sayer Al-Azzam.

**Investigation:** Reema Karasneh, Sarah Al Sharie, Sayer Al-Azzam, Shoroq M. Altawalbeh, Basheer Khassawneh, Muna Talafha, Mohammad Nusair, Abdel-Hameed Al-Mistarehi, Othman Beni Yonis, Mousa A. Al-Omary, Suad Kabbaha, Mamoon A. Aldeyab.

**Methodology:** Reema Karasneh, Sarah Al Sharie, Sayer Al-Azzam, Shoroq M. Altawalbeh, Basheer Khassawneh, Muna Talafha, Mohammad Nusair, Abdel-Hameed Al-Mistarehi, Othman Beni Yonis, Suad Kabbaha, Mamoon A. Aldeyab.

**Project administration:** Reema Karasneh, Sarah Al Sharie, Sayer Al-Azzam, Abdel-Hameed Al-Mistarehi, Othman Beni Yonis, Mousa A. Al-Omary, Mamoon A. Aldeyab.

**Resources:** Sayer Al-Azzam, Basheer Khassawneh, Abdel-Hameed Al-Mistarehi.

**Software:** Shoroq M. Altawalbeh.

**Supervision:** Sayer Al-Azzam, Othman Beni Yonis, Mousa A. Al-Omary, Mamoon A. Aldeyab.

**Validation:** Reema Karasneh, Sayer Al-Azzam, Shoroq M. Altawalbeh, Basheer Khassawneh, Mamoon A. Aldeyab.

**Visualization:** Reema Karasneh, Shoroq M. Altawalbeh, Basheer Khassawneh, Muna Talafha, Mohammad Nusair, Abdel-Hameed Al-Mistarehi, Othman Beni Yonis, Mamoon A. Aldeyab.

**Writing – original draft:** Reema Karasneh, Sarah Al Sharie.

**Writing – review & editing:** Reema Karasneh, Sarah Al Sharie, Sayer Al-Azzam, Shoroq M. Altawalbeh, Basheer Khassawneh, Muna Talafha, Mohammad Nusair, Abdel-Hameed Al-Mistarehi, Othman Beni Yonis, Mousa A. Al-Omary, Suad Kabbaha, Mamoon A. Aldeyab.

## Supplementary Material






