# Supplementary material for: Reported long-term effects of COVID-19 patients after hospital discharge in Jordan
Source: Medicine (Baltimore). 2023 Sep 22;102(38):e34633. doi: 10.1097/MD.0000000000034633 (PMC10519471; doi:10.1097/MD.0000000000034633)
Supplement: Supplementary file 1 [file medi-102-e34633-s001.docx]

**Self-reported symptoms questionnaire**

| **Date of interview: Time:** | | |
| --- | --- | --- |
| **Demographics:** | | |
| **المعلومات الديموغرافية:** | | |
| **Gender** | 🞎 Male 🞎 Female | |
| 🞎 ذكر 🞎 أنثى | | **الجنس** |
| **Age (years)** | **_ _ _ _ _ _ _ _** | |
| **_ _ _ _ _ _ _ _** | | **العمر (بالسنوات)** |
| **Marital status** | 🞎 Single 🞎 Married 🞎 Divorced 🞎 Widower | |
| 🞎 أعزب 🞎 متزوج 🞎 مطلق 🞎 ارمل | | **الحالة الإجتماعية** |
| **Educational level (Degree)** | 🞎 College or higher 🞎 High school or lower | |
| 🞎 بكالوريوس او اعلى 🞎 المرحلة الثانوية او اقل | | **أعلى درجة علمية؟** |
| **Employment** | 🞎 Employed 🞎 Unemployed 🞎 Retired | |
| 🞎 على رأس عملي 🞎 لا أعمل 🞎 متقاعد | | **الوضع الوظيفي** |
| **Nationality** | 🞎 Jordanian 🞎 Non-Jordanian | |
| 🞎 أردنية 🞎 غير أردنية | | **الجنسية** |
| **Smoking status** | 🞎 Current smoker (cigarettes, e-cigarettes, Shisha (Argila)  🞎 Ex-smoker (cigarettes, e-cigarettes, Shisha (Argila)  🞎 Not a smoker | |
| 🞎 مدخن (سجائر، سجائر الكترونية، ارجيلة)  🞎 مدخن سابق (سجائر، سجائر الكترونية، ارجيلة)  🞎 غير مدخن | | **التدخين** |

.

| **Vaccination info:** | | |
| --- | --- | --- |
| **معلومات المطعوم:** | | |
| **Were you vaccinated for COVID-19?** | 🞎 Yes 🞎 No | |
| 🞎 نعم 🞎 لا | | **هل أخذت مطعوم كوفيد-19؟** |
| **When did you receive your COVID-19 vaccine?** | 🞎 Before admission 🞎 After admission | |
| 🞎 قبل دخول المستشفى 🞎 بعد دخول المستشفى | | **متى أخذت مطعوم كوفيد-19؟** |

| **After discharge:** | |
| --- | --- |
| **بعد الخروج من المستشفى** | |
| Have you been discharged home on oxygen? | 🞎 Yes 🞎 No |
| 🞎 نعم 🞎 لا | هل خرجت إلى المنزل على الأكسجين؟ |
| If yes, for how long? |  |
|  | إذا كانت الإجابة بنعم ، كم المدة؟ |
| Have you been reinfected with COVID-19 after discharge? | 🞎 Yes 🞎 No |
| 🞎 نعم 🞎 لا | هل أصبت بفايروس كورونا مرة أخرى بعد الخروج من المستشفى؟ |
| Date of reinfection |  |
|  | تاريخ الإصابة الثانية |
| Have you been admitted to hospital for the reinfection? | 🞎 Yes 🞎 No |
|  | هل احتجت للدخول للمستشفى بعد الإصابة الثانية؟ |
| 🞎 نعم 🞎 لا |  |

| 1. **Chronic Diseases:** | | |
| --- | --- | --- |
| **الأمراض المزمنة** | | |
| **Have you suffered of any chronic diseases after discharge?** | 🞎 Yes 🞎 No | |
| 🞎 نعم 🞎 لا | | **هل تمت اصابتك باي مرض مزمن بعد خروجك من المستشفى؟** |
| **What are the chronic diseases you developed?** | 🞎 Hypertension 🞎 DM  🞎 Cardiovascular diseases 🞎 Cerebrovascular diseases 🞎 Other | |
| 🞎 ارتفاع ضغط الدم 🞎 سكري  🞎 أمراض قلبية مزمنة 🞎 أمراض عصبية مزمنة  🞎 غير ذلك | | **ما هي الأمراض المزمنة التي ظهرت بعد خروجك من المستشفى؟** |

| **In case of Diabetes before infection, did the control over your disease change after discharge?** | 🞎 Better control 🞎 Worse control  🞎 Same as before 🞎 I don’t have diabetes 🞎 Got diagnosed after discharge | |
| --- | --- | --- |
| 🞎 تحكم افضل 🞎 تحكم اسوأ  🞎لا يوجد تغيير 🞎لا اعاني من السكري 🞎تشخصت بالسكري بعد الخروج من المستشفى | | **في حالة الاصابة ب السكري من قبل اصابة كورونا, هل شعرت بأي اضطرابات في تحكمك بقراات سكر الدم؟** |

| **In case of Hypertension before infection, did the control over your disease change after discharge?** | 🞎 Better control 🞎 Worse control  🞎 Same as before 🞎 I don’t have HTN 🞎 Got diagnosed after discharge | |
| --- | --- | --- |
| 🞎 تحكم افضل 🞎 تحكم اسوأ  🞎لا يوجد تغيير 🞎 لا اعاني من ارتفاع ضغط الدم 🞎تشخصت بارتفاع ضغط الدم بعد الخروج من المستشفى | | **في حالة الاصابة ب ارتفاع ضغط الدم من قبل اصابة كورونا, هل شعرت بأي اضطرابات في تحكمك بقراات ضغط الدم؟** |

| 1. **Extreme fatigue:** | | |
| --- | --- | --- |
| **التعب الشديد:** | | |
| **Have you experienced extreme fatigue after being discharge from the hospital?** | 🞎 Yes 🞎 No | |
| 🞎 نعم 🞎 لا | | **هل عانيت من التعب الشديد بعد خروجك من المستشفى؟** |
| **When was the extreme fatigue experienced?** | 🞎 During COVID-19 infection  🞎 Before COVID-19 infection  🞎 Experienced for the first time after discharge | |
| 🞎 خلال فترة الإصابة بعدوى كوفيد-19  🞎 قبل الإصابة بعدوى كوفيد-19  🞎 شعرت بها للمرة الاولى بعد الخروج من المستشفى | | **متى عانيت من التعب الشديد؟** |
| **For how long have you experienced extreme fatigue?** | 🞎 4 weeks or less from the onset of COVID-19  🞎 4-12 weeks after the onset of COVID-19  🞎 More than 12 weeks after the onset of COVID-19 | |
| 🞎 4 اسابيع او اقل منذ بداية العدوى  🞎 4-12 اسبوع منذ بداية العدوى  🞎 أكثر من 12 اسبوع منذ بداية العدوى | | **لكم من الوقت عانيت من التعب الشديد؟** |
| **If you have experienced extreme fatigue before COVID-19 infection, how it is now?** | 🞎 Same as before  🞎 Worse than before  🞎 Better than before  🞎 Completely resolved | |
| 🞎 كما كانت من قبل  🞎 أسوأ من قبل  🞎 أفضل من قبل  🞎 اختفت تماما | | **اذا عانيت من التعب الشديد , كيف تصفه الان؟** |

| 1. **Cough:** | | |
| --- | --- | --- |
| **السعال:** | | |
| **Have you experienced cough after being discharge from the hospital?** | 🞎 Yes 🞎 No | |
| 🞎 نعم 🞎 لا | | **هل عانيت من السعال بعد خروجك من المستشفى؟** |
| **When was the cough experienced?** | 🞎 During COVID-19 infection  🞎 Before COVID-19 infection  🞎 Experienced for the first time after discharge | |
| 🞎 خلال فترة الإصابة بعدوى كوفيد-19  🞎 قبل الإصابة بعدوى كوفيد-19  🞎 شعرت بها للمرة الاولى بعد الخروج من المستشفى | | **متى عانيت من السعال؟** |
| **For how long have you experienced cough?** | 🞎 4 weeks or less from the onset of COVID-19  🞎 4-12 weeks after the onset of COVID-19  🞎 More than 12 weeks after the onset of COVID-19 | |
| 🞎 4 اسابيع او اقل منذ بداية العدوى  🞎 4-12 اسبوع منذ بداية العدوى  🞎 أكثر من 12 اسبوع منذ بداية العدوى | | **لكم من الوقت عانيت من السعال؟** |
| **If you have experienced cough before COVID-19 infection, how it is now?** | 🞎 Same as before  🞎 Worse than before  🞎 Better than before  🞎 Completely resolved | |
| 🞎 كما كانت من قبل  🞎 أسوأ من قبل  🞎 أفضل من قبل  🞎 اختفى تماما | | **اذا عانيت من السعال , كيف تصفه الان؟** |

| 1. **Hemoptysis:** | | |
| --- | --- | --- |
| **دم مع السعال** | | |
| **Have you experienced hemoptysis after being discharge from the hospital?** | 🞎 Yes 🞎 No | |
| 🞎 نعم 🞎 لا | | **هل عانيت من دم مع السعال بعد خروجك من المستشفى؟** |
| **When was the hemoptysis experienced?** | 🞎 During COVID-19 infection  🞎 Before COVID-19 infection  🞎 Experienced for the first time after discharge | |
| 🞎 خلال فترة الإصابة بعدوى كوفيد-19  🞎 قبل الإصابة بعدوى كوفيد-19  🞎 شعرت بها للمرة الاولى بعد الخروج من المستشفى | | **متى عانيت من دم مع السعال؟** |
| **For how long have you experienced hemoptysis?** | 🞎 4 weeks or less from the onset of COVID-19  🞎 4-12 weeks after the onset of COVID-19  🞎 More than 12 weeks after the onset of COVID-19 | |
| 🞎 4 اسابيع او اقل منذ بداية العدوى  🞎 4-12 اسبوع منذ بداية العدوى  🞎 أكثر من 12 اسبوع منذ بداية العدوى | | **لكم من الوقت عانيت من دم مع السعال؟** |
| **If you have experienced hemoptysis before COVID-19 infection, how it is now?** | 🞎 Same as before  🞎 Worse than before  🞎 Better than before  🞎 Completely resolved | |
| 🞎 كما كانت من قبل  🞎 أسوأ من قبل  🞎 أفضل من قبل  🞎 اختفت تماما | | **اذا عانيت من دم مع السعال , كيف تصفه الان؟** |

| 1. **Sputum Production:** | | |
| --- | --- | --- |
| **افرازات بلغمية:** | | |
| **Have you experienced sputum production after being discharge from the hospital?** | 🞎 Yes 🞎 No | |
| 🞎 نعم 🞎 لا | | **هل عانيت من افرازات بلغمية بعد خروجك من المستشفى؟** |
| **When was the sputum production experienced?** | 🞎 During COVID-19 infection  🞎 Before COVID-19 infection  🞎 Experienced for the first time after discharge | |
| 🞎 خلال فترة الإصابة بعدوى كوفيد-19  🞎 قبل الإصابة بعدوى كوفيد-19  🞎 شعرت بها للمرة الاولى بعد الخروج من المستشفى | | **متى عانيت من افرازات بلغمية؟** |
| **For how long have you experienced sputum production?** | 🞎 4 weeks or less from the onset of COVID-19  🞎 4-12 weeks after the onset of COVID-19  🞎 More than 12 weeks after the onset of COVID-19 | |
| 🞎 4 اسابيع او اقل منذ بداية العدوى  🞎 4-12 اسبوع منذ بداية العدوى  🞎 أكثر من 12 اسبوع منذ بداية العدوى | | **لكم من الوقت عانيت من افرازات بلغمية؟** |
| **If you have experienced sputum production before COVID-19 infection, how it is now?** | 🞎 Same as before  🞎 Worse than before  🞎 Better than before  🞎 Completely resolved | |
| 🞎 كما كانت من قبل  🞎 أسوأ من قبل  🞎 أفضل من قبل  🞎 اختفت تماما | | **اذا عانيت من افرازات بلغمية , كيف تصفها الان؟** |

| 1. **Low grade fever (37-38):** | | |
| --- | --- | --- |
| **الحمى المنخفضة (37-38):** | | |
| **Have you experienced low grade fever after being discharge from the hospital?** | 🞎 Yes 🞎 No | |
| 🞎 نعم 🞎 لا | | **هل عانيت من الحمى المنخفضة بعد خروجك من المستشفى؟** |
| **For how long have you experienced low grade fever?** | 🞎 4 weeks or less from the onset of COVID-19  🞎 4-12 weeks after the onset of COVID-19  🞎 More than 12 weeks after the onset of COVID-19 | |
| 🞎 4 اسابيع او اقل منذ بداية العدوى  🞎 4-12 اسبوع منذ بداية العدوى  🞎 أكثر من 12 اسبوع منذ بداية العدوى | | **لكم من الوقت عانيت من الحمى المنخفضة؟** |
| **When was the low-grade fever experienced?** | 🞎 During COVID-19 infection  🞎 Before COVID-19 infection  🞎 Experienced for the first time after discharge | |
| 🞎 خلال فترة الإصابة بعدوى كوفيد-19  🞎 قبل الإصابة بعدوى كوفيد-19  🞎 شعرت بها للمرة الاولى بعد الخروج من المستشفى | | **متى عانيت من الحمى المنخفضة ؟** |
| **If you have experienced low-grade fever before COVID-19 infection, how it is now?** | 🞎 Same as before  🞎 Worse than before  🞎 Better than before  🞎 Completely resolved | |
| 🞎 كما كانت من قبل  🞎 أسوأ من قبل  🞎 أفضل من قبل  🞎 اختفت تماما | | **اذا عانيت من الحمى المنخفضة , كيف تصفها الان؟** |

| 1. **High grade fever (>38):** | | |
| --- | --- | --- |
| **الحمى المرتفعة (>38):** | | |
| **Have you experienced high grade fever after being discharge from the hospital?** | 🞎 Yes 🞎 No | |
| 🞎 نعم 🞎 لا | | **هل عانيت من الحمى المرتفعة بعد خروجك من المستشفى؟** |
| **For how long have you experienced high grade fever?** | 🞎 4 weeks or less from the onset of COVID-19  🞎 4-12 weeks after the onset of COVID-19  🞎 More than 12 weeks after the onset of COVID-19 | |
| 🞎 4 اسابيع او اقل منذ بداية العدوى  🞎 4-12 اسبوع منذ بداية العدوى  🞎 أكثر من 12 اسبوع منذ بداية العدوى | | **لكم من الوقت عانيت من الحمى المرتفعة؟** |
| **When was the high-grade fever experienced?** | 🞎 During COVID-19 infection  🞎 Before COVID-19 infection  🞎 Experienced for the first time after discharge | |
| 🞎 خلال فترة الإصابة بعدوى كوفيد-19  🞎 قبل الإصابة بعدوى كوفيد-19  🞎 شعرت بها للمرة الاولى بعد الخروج من المستشفى | | **متى عانيت من الحمى المرتفعة ؟** |
| **If you have experienced high-grade fever before COVID-19 infection, how it is now?** | 🞎 Same as before  🞎 Worse than before  🞎 Better than before  🞎 Completely resolved | |
| 🞎 كما كانت من قبل  🞎 أسوأ من قبل  🞎 أفضل من قبل  🞎 اختفت تماما | | **اذا عانيت من الحمى المرتفعة , كيف تصفها الان؟** |

| 1. **Chills:** | | |
| --- | --- | --- |
| **قشعريرة:** | | |
| **Have you experienced chills after being discharge from the hospital?** | 🞎 Yes 🞎 No | |
| 🞎 نعم 🞎 لا | | **هل عانيت من قشعريرة بعد خروجك من المستشفى؟** |
| **When was the chills experienced?** | 🞎 During COVID-19 infection  🞎 Before COVID-19 infection  🞎 Experienced for the first time after discharge | |
| 🞎 خلال فترة الإصابة بعدوى كوفيد-19  🞎 قبل الإصابة بعدوى كوفيد-19  🞎 شعرت بها للمرة الاولى بعد الخروج من المستشفى | | **متى عانيت من قشعريرة؟** |
| **For how long have you experienced chills?** | 🞎 4 weeks or less from the onset of COVID-19  🞎 4-12 weeks after the onset of COVID-19  🞎 More than 12 weeks after the onset of COVID-19 | |
| 🞎 4 اسابيع او اقل منذ بداية العدوى  🞎 4-12 اسبوع منذ بداية العدوى  🞎 أكثر من 12 اسبوع منذ بداية العدوى | | **لكم من الوقت عانيت من قشعريرة؟** |
| **If you have experienced chills before COVID-19 infection, how it is now?** | 🞎 Same as before  🞎 Worse than before  🞎 Better than before  🞎 Completely resolved | |
| 🞎 كما كانت من قبل  🞎 أسوأ من قبل  🞎 أفضل من قبل  🞎 اختفى تماما | | **اذا عانيت من قشعريرة , كيف تصفها الان؟** |

| 1. **Nasal congestion / Runny nose:** | | |
| --- | --- | --- |
| **احتقان او سيلان في الانف:** | | |
| **Have you experienced Nasal congestion / Runny nose after being discharge from the hospital?** | 🞎 Yes 🞎 No | |
| 🞎 نعم 🞎 لا | | **هل عانيت من احتقان او سيلان في الانف بعد خروجك من المستشفى؟** |
| **When was the Nasal congestion / Runny nose experienced?** | 🞎 During COVID-19 infection  🞎 Before COVID-19 infection  🞎 Experienced for the first time after discharge | |
| 🞎 خلال فترة الإصابة بعدوى كوفيد-19  🞎 قبل الإصابة بعدوى كوفيد-19  🞎 شعرت بها للمرة الاولى بعد الخروج من المستشفى | | **متى عانيت من احتقان او سيلان في الانف؟** |
| **For how long have you experienced Nasal congestion / Runny nose?** | 🞎 4 weeks or less from the onset of COVID-19  🞎 4-12 weeks after the onset of COVID-19  🞎 More than 12 weeks after the onset of COVID-19 | |
| 🞎 4 اسابيع او اقل منذ بداية العدوى  🞎 4-12 اسبوع منذ بداية العدوى  🞎 أكثر من 12 اسبوع منذ بداية العدوى | | **لكم من الوقت عانيت من احتقان او سيلان في الانف؟** |
| **If you have experienced Nasal congestion / Runny nose before COVID-19 infection, how it is now?** | 🞎 Same as before  🞎 Worse than before  🞎 Better than before  🞎 Completely resolved | |
| 🞎 كما كانت من قبل  🞎 أسوأ من قبل  🞎 أفضل من قبل  🞎 اختفى تماما | | **اذا عانيت من احتقان او سيلان في الانف , كيف تصفه الان؟** |

| 1. **Nose bleed:** | | |
| --- | --- | --- |
| **نزيف من الانف:** | | |
| **Have you experienced Nose bleed after being discharge from the hospital?** | 🞎 Yes 🞎 No | |
| 🞎 نعم 🞎 لا | | **هل عانيت من نزيف من الانف بعد خروجك من المستشفى؟** |
| **When was the Nose bleed experienced?** | 🞎 During COVID-19 infection  🞎 Before COVID-19 infection  🞎 Experienced for the first time after discharge | |
| 🞎 خلال فترة الإصابة بعدوى كوفيد-19  🞎 قبل الإصابة بعدوى كوفيد-19  🞎 شعرت بها للمرة الاولى بعد الخروج من المستشفى | | **متى عانيت من نزيف من الانف؟** |
| **For how long have you experienced Nose bleed?** | 🞎 4 weeks or less from the onset of COVID-19  🞎 4-12 weeks after the onset of COVID-19  🞎 More than 12 weeks after the onset of COVID-19 | |
| 🞎 4 اسابيع او اقل منذ بداية العدوى  🞎 4-12 اسبوع منذ بداية العدوى  🞎 أكثر من 12 اسبوع منذ بداية العدوى | | **لكم من الوقت عانيت من نزيف من الانف؟** |
| **If you have experienced Nose bleed before COVID-19 infection, how it is now?** | 🞎 Same as before  🞎 Worse than before  🞎 Better than before  🞎 Completely resolved | |
| 🞎 كما كانت من قبل  🞎 أسوأ من قبل  🞎 أفضل من قبل  🞎 اختفى تماما | | **اذا عانيت من نزيف من الانف , كيف تصفه الان؟** |

| 1. **Dyspnea:** | | |
| --- | --- | --- |
| **ضيق في التنفس:** | | |
| **Have you experienced dyspnea after being discharge from the hospital?** | 🞎 Yes 🞎 No | |
| 🞎 نعم 🞎 لا | | **هل عانيت من ضيق في التنفس بعد خروجك من المستشفى؟** |
| **When was the dyspnea experienced?** | 🞎 During COVID-19 infection  🞎 Before COVID-19 infection  🞎 Experienced for the first time after discharge | |
| 🞎 خلال فترة الإصابة بعدوى كوفيد-19  🞎 قبل الإصابة بعدوى كوفيد-19  🞎 شعرت بها للمرة الاولى بعد الخروج من المستشفى | | **متى عانيت من ضيق في التنفس؟** |
| **For how long have you experienced dyspnea?** | 🞎 4 weeks or less from the onset of COVID-19  🞎 4-12 weeks after the onset of COVID-19  🞎 More than 12 weeks after the onset of COVID-19 | |
| 🞎 4 اسابيع او اقل منذ بداية العدوى  🞎 4-12 اسبوع منذ بداية العدوى  🞎 أكثر من 12 اسبوع منذ بداية العدوى | | **لكم من الوقت عانيت من ضيق في التنفس؟** |
| **If you have experienced dyspnea before COVID-19 infection, how it is now?** | 🞎 Same as before  🞎 Worse than before  🞎 Better than before  🞎 Completely resolved | |
| 🞎 كما كانت من قبل  🞎 أسوأ من قبل  🞎 أفضل من قبل  🞎 اختفى تماما | | **اذا عانيت من ضيق في التنفس , كيف تصفه الان؟** |

| **mMRC (Modified Medical Research Council) Dyspnea Scale:** | | **Score:** |
| --- | --- | --- |
| 🞎 Dyspnea only with strenuous exercise  🞎 Dyspnea when hurrying or walking up a slight hill  🞎 Walks slower than people of the same age because of dyspnea or has to stop for breath when walking at own pace  🞎 Stops for breath after walking 100 yards (91 m) or after a few minutes  🞎 Too dyspneic to leave house or breathless when dressing | 🞎 ضيق نفس فقط مع التمارين الشاقّة  🞎 ضيق نفس مع الاسراع او المشي صعودا  🞎 تمشي ابطأ من الاشخاص من نفس الفئة العمريّة بسبب ضيق النفس او تحتاج الى التوقف لاخذ نفس عندما تمشي على وتيرتك الاعتياديّة  🞎 تتوقّف لاخذ نفس بعد المشي لمسافة 91 متر او بعد عدّة دقائق  🞎 لا تستطيع الخروج من المنزل او ارتداء ملابسك بسبب ضيق التنفّس | |

| 1. **Sore throat:** | | |
| --- | --- | --- |
| **احتقان في الحلق:** | | |
| **Have you experienced sore throat after being discharge from the hospital?** | 🞎 Yes 🞎 No | |
| 🞎 نعم 🞎 لا | | **هل عانيت من احتقان في الحلق بعد خروجك من المستشفى؟** |
| **For how long have you experienced sore throat?** | 🞎 4 weeks or less from the onset of COVID-19  🞎 4-12 weeks after the onset of COVID-19  🞎 More than 12 weeks after the onset of COVID-19 | |
| 🞎 4 اسابيع او اقل منذ بداية العدوى  🞎 4-12 اسبوع منذ بداية العدوى  🞎 أكثر من 12 اسبوع منذ بداية العدوى | | **لكم من الوقت عانيت من احتقان في الحلق؟** |
| **When was the sore throat experienced?** | 🞎 During COVID-19 infection  🞎 Before COVID-19 infection  🞎 Experienced for the first time after discharge | |
| 🞎 خلال فترة الإصابة بعدوى كوفيد-19  🞎 قبل الإصابة بعدوى كوفيد-19  🞎 شعرت بها للمرة الاولى بعد الخروج من المستشفى | | **متى عانيت من احتقان في الحلق ؟** |
| **If you have experienced a sore throat before COVID-19 infection, how it is now?** | 🞎 Same as before  🞎 Worse than before  🞎 Better than before  🞎 Completely resolved | |
| 🞎 كما كانت من قبل  🞎 أسوأ من قبل  🞎 أفضل من قبل  🞎 اختفى تماما | | **اذا عانيت من احتقان في الحلق , كيف تصفه الان؟** |

| 1. **Chest pain:** | | |
| --- | --- | --- |
| **الم في الصدر:** | | |
| **Have you experienced chest pain after being discharge from the hospital?** | 🞎 Yes 🞎 No | |
| 🞎 نعم 🞎 لا | | **هل عانيت من الم في الصدر بعد خروجك من المستشفى؟** |
| **When was the chest pain experienced?** | 🞎 During COVID-19 infection  🞎 Before COVID-19 infection  🞎 Experienced for the first time after discharge | |
| 🞎 خلال فترة الإصابة بعدوى كوفيد-19  🞎 قبل الإصابة بعدوى كوفيد-19  🞎 شعرت بها للمرة الاولى بعد الخروج من المستشفى | | **متى عانيت من الم في الصدر؟** |
| **For how long have you experienced chest pain?** | 🞎 4 weeks or less from the onset of COVID-19  🞎 4-12 weeks after the onset of COVID-19  🞎 More than 12 weeks after the onset of COVID-19 | |
| 🞎 4 اسابيع او اقل منذ بداية العدوى  🞎 4-12 اسبوع منذ بداية العدوى  🞎 أكثر من 12 اسبوع منذ بداية العدوى | | **لكم من الوقت عانيت من الم في الصدر؟** |
| **If you have experienced chest pain before COVID-19 infection, how it is now?** | 🞎 Same as before  🞎 Worse than before  🞎 Better than before  🞎 Completely resolved | |
| 🞎 كما كانت من قبل  🞎 أسوأ من قبل  🞎 أفضل من قبل  🞎 اختفى تماما | | **اذا عانيت من الم في الصدر , كيف تصفه الان؟** |

| 1. **Palpitation:** | | |
| --- | --- | --- |
| **خفقان في القلب:** | | |
| **Have you experienced palpitation after being discharge from the hospital?** | 🞎 Yes 🞎 No | |
| 🞎 نعم 🞎 لا | | **هل عانيت من خفقان في القلب بعد خروجك من المستشفى؟** |
| **When was the palpitation experienced?** | 🞎 During COVID-19 infection  🞎 Before COVID-19 infection  🞎 Experienced for the first time after discharge | |
| 🞎 خلال فترة الإصابة بعدوى كوفيد-19  🞎 قبل الإصابة بعدوى كوفيد-19  🞎 شعرت بها للمرة الاولى بعد الخروج من المستشفى | | **متى عانيت من خفقان في القلب؟** |
| **For how long have you experienced palpitation?** | 🞎 4 weeks or less from the onset of COVID-19  🞎 4-12 weeks after the onset of COVID-19  🞎 More than 12 weeks after the onset of COVID-19 | |
| 🞎 4 اسابيع او اقل منذ بداية العدوى  🞎 4-12 اسبوع منذ بداية العدوى  🞎 أكثر من 12 اسبوع منذ بداية العدوى | | **لكم من الوقت عانيت من خفقان في القلب؟** |
| **If you have experienced palpitation before COVID-19 infection, how it is now?** | 🞎 Same as before  🞎 Worse than before  🞎 Better than before  🞎 Completely resolved | |
| 🞎 كما كانت من قبل  🞎 أسوأ من قبل  🞎 أفضل من قبل  🞎 اختفى تماما | | **اذا عانيت من خفقان في القلب , كيف تصفه الان؟** |

| 1. **Changes in mood (anxiety/depression):** | | |
| --- | --- | --- |
| **تقلبات مزاجيّة (قلق/ اكتئاب):** | | |
| **Have you experienced changes in mood (anxiety/depression) after being discharge from the hospital?** | 🞎 Yes 🞎 No | |
| 🞎 نعم 🞎 لا | | **هل عانيت من تقلبات مزاجيّة (قلق/ اكتئاب) بعد خروجك من المستشفى؟** |
| **When was the changes in mood (anxiety/depression) experienced?** | 🞎 During COVID-19 infection  🞎 Before COVID-19 infection  🞎 Experienced for the first time after discharge | |
| 🞎 خلال فترة الإصابة بعدوى كوفيد-19  🞎 قبل الإصابة بعدوى كوفيد-19  🞎 شعرت بها للمرة الاولى بعد الخروج من المستشفى | | **متى عانيت من تقلبات مزاجيّة (قلق/ اكتئاب)؟** |
| **For how long have you experienced changes in mood (anxiety/depression)?** | 🞎 4 weeks or less from the onset of COVID-19  🞎 4-12 weeks after the onset of COVID-19  🞎 More than 12 weeks after the onset of COVID-19 | |
| 🞎 4 اسابيع او اقل منذ بداية العدوى  🞎 4-12 اسبوع منذ بداية العدوى  🞎 أكثر من 12 اسبوع منذ بداية العدوى | | **لكم من الوقت عانيت من تقلبات مزاجيّة (قلق/ اكتئاب)؟** |
| **If you have experienced changes in mood (anxiety/depression) before COVID-19 infection, how it is now?** | 🞎 Same as before  🞎 Worse than before  🞎 Better than before  🞎 Completely resolved | |
| 🞎 كما كانت من قبل  🞎 أسوأ من قبل  🞎 أفضل من قبل  🞎 اختفت تماما | | **اذا عانيت من تقلبات مزاجيّة (قلق/ اكتئاب) , كيف تصفها الان؟** |

| 1. **Headache:** | | |
| --- | --- | --- |
| **الصداع:** | | |
| **Have you experienced Headache after being discharge from the hospital?** | 🞎 Yes 🞎 No | |
| 🞎 نعم 🞎 لا | | **هل عانيت من الصداع بعد خروجك من المستشفى؟** |
| **When was the Headache experienced?** | 🞎 During COVID-19 infection  🞎 Before COVID-19 infection  🞎 Experienced for the first time after discharge | |
| 🞎 خلال فترة الإصابة بعدوى كوفيد-19  🞎 قبل الإصابة بعدوى كوفيد-19  🞎 شعرت بها للمرة الاولى بعد الخروج من المستشفى | | **متى عانيت من الصداع؟** |
| **For how long have you experienced Headache?** | 🞎 4 weeks or less from the onset of COVID-19  🞎 4-12 weeks after the onset of COVID-19  🞎 More than 12 weeks after the onset of COVID-19 | |
| 🞎 4 اسابيع او اقل منذ بداية العدوى  🞎 4-12 اسبوع منذ بداية العدوى  🞎 أكثر من 12 اسبوع منذ بداية العدوى | | **لكم من الوقت عانيت من الصداع؟** |
| **If you have experienced Headache before COVID-19 infection, how it is now?** | 🞎 Same as before  🞎 Worse than before  🞎 Better than before  🞎 Completely resolved | |
| 🞎 كما كانت من قبل  🞎 أسوأ من قبل  🞎 أفضل من قبل  🞎 اختفى تماما | | **اذا عانيت من الصداع , كيف تصفه الان؟** |

| 1. **Seizures:** | | |
| --- | --- | --- |
| **نوبات صرع:** | | |
| **Have you experienced Seizures after being discharge from the hospital?** | 🞎 Yes 🞎 No | |
| 🞎 نعم 🞎 لا | | **هل عانيت من نوبات صرع بعد خروجك من المستشفى؟** |
| **When was the Seizures experienced?** | 🞎 During COVID-19 infection  🞎 Before COVID-19 infection  🞎 Experienced for the first time after discharge | |
| 🞎 خلال فترة الإصابة بعدوى كوفيد-19  🞎 قبل الإصابة بعدوى كوفيد-19  🞎 شعرت بها للمرة الاولى بعد الخروج من المستشفى | | **متى عانيت من نوبات صرع؟** |
| **For how long have you experienced Seizures?** | 🞎 4 weeks or less from the onset of COVID-19  🞎 4-12 weeks after the onset of COVID-19  🞎 More than 12 weeks after the onset of COVID-19 | |
| 🞎 4 اسابيع او اقل منذ بداية العدوى  🞎 4-12 اسبوع منذ بداية العدوى  🞎 أكثر من 12 اسبوع منذ بداية العدوى | | **لكم من الوقت عانيت من نوبات صرع؟** |
| **If you have experienced Seizures before COVID-19 infection, how it is now?** | 🞎 Same as before  🞎 Worse than before  🞎 Better than before  🞎 Completely resolved | |
| 🞎 كما كانت من قبل  🞎 أسوأ من قبل (خلل لكن ليس فقدان تام)  🞎 أفضل من قبل  🞎 اختفى تماما | | **اذا عانيت من نوبات صرع , كيف تصفها الان؟** |

| 1. **Loss of taste:** | | |
| --- | --- | --- |
| **فقدان حاسة التذوق:** | | |
| **Have you experienced loss of taste after being discharge from the hospital?** | 🞎 Yes 🞎 No | |
| 🞎 نعم 🞎 لا | | **هل عانيت من فقدان حاسة التذوق بعد خروجك من المستشفى؟** |
| **For how long have you experienced loss of taste?** | 🞎 4 weeks or less from the onset of COVID-19  🞎 4-12 weeks after the onset of COVID-19  🞎 More than 12 weeks after the onset of COVID-19 | |
| 🞎 4 اسابيع او اقل منذ بداية العدوى  🞎 4-12 اسبوع منذ بداية العدوى  🞎 أكثر من 12 اسبوع منذ بداية العدوى | | **لكم من الوقت عانيت من فقدان حاسة التذوق؟** |
| **When was the loss of taste experienced?** | 🞎 During COVID-19 infection  🞎 Before COVID-19 infection  🞎 Experienced for the first time after discharge | |
| 🞎 خلال فترة الإصابة بعدوى كوفيد-19  🞎 قبل الإصابة بعدوى كوفيد-19  🞎 شعرت بها للمرة الاولى بعد الخروج من المستشفى | | **متى عانيت من فقدان حاسة التذوق ؟** |
| **If you have experienced loss of taste before COVID-19 infection, how it is now?** | 🞎 Same as before  🞎 Worse than before  🞎 Better than before  🞎 Completely resolved | |
| 🞎 كما كانت من قبل  🞎 أسوأ من قبل (خلل لكن ليس فقدان تام)  🞎 أفضل من قبل  🞎 اختفت تماما | | **اذا عانيت من فقدان حاسة التذوق , كيف تصفها الان؟** |

| 1. **Loss of smell:** | | |
| --- | --- | --- |
| **فقدان حاسة الشم:** | | |
| **Have you experienced loss of smell after being discharge from the hospital?** | 🞎 Yes 🞎 No | |
| 🞎 نعم 🞎 لا | | **هل عانيت من فقدان حاسة الشم بعد خروجك من المستشفى؟** |
| **For how long have you experienced loss of smell?** | 🞎 4 weeks or less from the onset of COVID-19  🞎 4-12 weeks after the onset of COVID-19  🞎 More than 12 weeks after the onset of COVID-19 | |
| 🞎 4 اسابيع او اقل منذ بداية العدوى  🞎 4-12 اسبوع منذ بداية العدوى  🞎 أكثر من 12 اسبوع منذ بداية العدوى | | **لكم من الوقت عانيت من فقدان حاسة الشم؟** |
| **When was the loss of smell experienced?** | 🞎 During COVID-19 infection  🞎 Before COVID-19 infection  🞎 Experienced for the first time after discharge | |
| 🞎 خلال فترة الإصابة بعدوى كوفيد-19  🞎 قبل الإصابة بعدوى كوفيد-19  🞎 شعرت بها للمرة الاولى بعد الخروج من المستشفى | | **متى عانيت من فقدان حاسة الشم ؟** |
| **If you have experienced loss of smell before COVID-19 infection, how it is now?** | 🞎 Same as before  🞎 Worse than before  🞎 Better than before  🞎 Completely resolved | |
| 🞎 كما كانت من قبل  🞎 أسوأ من قبل  🞎 أفضل من قبل  🞎 اختفت تماما | | **اذا عانيت من فقدان حاسة الشم , كيف تصفها الان؟** |

| 1. **Diarrhea:** | | |
| --- | --- | --- |
| **اسهال:** | | |
| **Have you experienced diarrhea after being discharge from the hospital?** | 🞎 Yes 🞎 No | |
| 🞎 نعم 🞎 لا | | **هل عانيت من اسهال بعد خروجك من المستشفى؟** |
| **For how long have you experienced diarrhea?** | 🞎 4 weeks or less from the onset of COVID-19  🞎 4-12 weeks after the onset of COVID-19  🞎 More than 12 weeks after the onset of COVID-19 | |
| 🞎 4 اسابيع او اقل منذ بداية العدوى  🞎 4-12 اسبوع منذ بداية العدوى  🞎 أكثر من 12 اسبوع منذ بداية العدوى | | **لكم من الوقت عانيت من اسهال؟** |
| **When was the diarrhea experienced?** | 🞎 During COVID-19 infection  🞎 Before COVID-19 infection  🞎 Experienced for the first time after discharge | |
| 🞎 خلال فترة الإصابة بعدوى كوفيد-19  🞎 قبل الإصابة بعدوى كوفيد-19  🞎 شعرت بها للمرة الاولى بعد الخروج من المستشفى | | **متى عانيت من اسهال ؟** |
| **If you have experienced diarrhea before COVID-19 infection, how it is now?** | 🞎 Same as before  🞎 Worse than before  🞎 Better than before  🞎 Completely resolved | |
| 🞎 كما كانت من قبل  🞎 أسوأ من قبل  🞎 أفضل من قبل  🞎 اختفى تماما | | **اذا عانيت من اسهال , كيف تصفه الان؟** |

| 1. **Vomiting:** | | |
| --- | --- | --- |
| **استفراغ:** | | |
| **Have you experienced vomiting after being discharge from the hospital?** | 🞎 Yes 🞎 No | |
| 🞎 نعم 🞎 لا | | **هل عانيت من استفراغ بعد خروجك من المستشفى؟** |
| **For how long have you experienced vomiting?** | 🞎 4 weeks or less from the onset of COVID-19  🞎 4-12 weeks after the onset of COVID-19  🞎 More than 12 weeks after the onset of COVID-19 | |
| 🞎 4 اسابيع او اقل منذ بداية العدوى  🞎 4-12 اسبوع منذ بداية العدوى  🞎 أكثر من 12 اسبوع منذ بداية العدوى | | **لكم من الوقت عانيت من استفراغ؟** |
| **When was the vomiting experienced?** | 🞎 During COVID-19 infection  🞎 Before COVID-19 infection  🞎 Experienced for the first time after discharge | |
| 🞎 خلال فترة الإصابة بعدوى كوفيد-19  🞎 قبل الإصابة بعدوى كوفيد-19  🞎 شعرت بها للمرة الاولى بعد الخروج من المستشفى | | **متى عانيت من استفراغ ؟** |
| **If you have experienced vomiting before COVID-19 infection, how it is now?** | 🞎 Same as before  🞎 Worse than before  🞎 Better than before  🞎 Completely resolved | |
| 🞎 كما كانت من قبل  🞎 أسوأ من قبل  🞎 أفضل من قبل  🞎 اختفى تماما | | **اذا عانيت من استفراغ , كيف تصفه الان؟** |

| 1. **Abdominal pain:** | | |
| --- | --- | --- |
| **الم في البطن:** | | |
| **Have you experienced abdominal pain after being discharge from the hospital?** | 🞎 Yes 🞎 No | |
| 🞎 نعم 🞎 لا | | **هل عانيت من الم في البطن بعد خروجك من المستشفى؟** |
| **For how long have you experienced abdominal pain?** | 🞎 4 weeks or less from the onset of COVID-19  🞎 4-12 weeks after the onset of COVID-19  🞎 More than 12 weeks after the onset of COVID-19 | |
| 🞎 4 اسابيع او اقل منذ بداية العدوى  🞎 4-12 اسبوع منذ بداية العدوى  🞎 أكثر من 12 اسبوع منذ بداية العدوى | | **لكم من الوقت عانيت من الم في البطن؟** |
| **When was the abdominal pain experienced?** | 🞎 During COVID-19 infection  🞎 Before COVID-19 infection  🞎 Experienced for the first time after discharge | |
| 🞎 خلال فترة الإصابة بعدوى كوفيد-19  🞎 قبل الإصابة بعدوى كوفيد-19  🞎 شعرت بها للمرة الاولى بعد الخروج من المستشفى | | **متى عانيت من الم في البطن ؟** |
| **If you have experienced abdominal pain before COVID-19 infection, how it is now?** | 🞎 Same as before  🞎 Worse than before  🞎 Better than before  🞎 Completely resolved | |
| 🞎 كما كانت من قبل  🞎 أسوأ من قبل  🞎 أفضل من قبل  🞎 اختفى تماما | | **اذا عانيت من الم في البطن , كيف تصفه الان؟** |

| 1. **Myalgia (muscles aches) or Arthralgia (joints pain):** | | |
| --- | --- | --- |
| **الم في العضلات او المفاصل:** | | |
| **Have you experienced Myalgia (muscles aches) or Arthralgia (joints pain) after being discharge from the hospital?** | 🞎 Yes 🞎 No | |
| 🞎 نعم 🞎 لا | | **هل عانيت من الم في العضلات او المفاصل بعد خروجك من المستشفى؟** |
| **For how long have you experienced Myalgia (muscles aches) or Arthralgia (joints pain)?** | 🞎 4 weeks or less from the onset of COVID-19  🞎 4-12 weeks after the onset of COVID-19  🞎 More than 12 weeks after the onset of COVID-19 | |
| 🞎 4 اسابيع او اقل منذ بداية العدوى  🞎 4-12 اسبوع منذ بداية العدوى  🞎 أكثر من 12 اسبوع منذ بداية العدوى | | **لكم من الوقت عانيت من الم في العضلات او المفاصل؟** |
| **When was the Myalgia (muscles aches) or Arthralgia (joints pain) experienced?** | 🞎 During COVID-19 infection  🞎 Before COVID-19 infection  🞎 Experienced for the first time after discharge | |
| 🞎 خلال فترة الإصابة بعدوى كوفيد-19  🞎 قبل الإصابة بعدوى كوفيد-19  🞎 شعرت بها للمرة الاولى بعد الخروج من المستشفى | | **متى عانيت من الم في العضلات او المفاصل ؟** |
| **If you have experienced Myalgia (muscles aches) or Arthralgia (joints pain) before COVID-19 infection, how it is now?** | 🞎 Same as before  🞎 Worse than before  🞎 Better than before  🞎 Completely resolved | |
| 🞎 كما كانت من قبل  🞎 أسوأ من قبل  🞎 أفضل من قبل  🞎 اختفى تماما | | **اذا عانيت من الم في العضلات او المفاصل , كيف تصفه الان؟** |

| 1. **Skin rash:** | | |
| --- | --- | --- |
| **حساسية على الجلد:** | | |
| **Have you experienced skin rash after being discharge from the hospital?** | 🞎 Yes 🞎 No | |
| 🞎 نعم 🞎 لا | | **هل عانيت من حساسية على الجلد بعد خروجك من المستشفى؟** |
| **For how long have you experienced skin rash?** | 🞎 4 weeks or less from the onset of COVID-19  🞎 4-12 weeks after the onset of COVID-19  🞎 More than 12 weeks after the onset of COVID-19 | |
| 🞎 4 اسابيع او اقل منذ بداية العدوى  🞎 4-12 اسبوع منذ بداية العدوى  🞎 أكثر من 12 اسبوع منذ بداية العدوى | | **لكم من الوقت عانيت من حساسية على الجلد؟** |
| **When was the skin rash experienced?** | 🞎 During COVID-19 infection  🞎 Before COVID-19 infection  🞎 Experienced for the first time after discharge | |
| 🞎 خلال فترة الإصابة بعدوى كوفيد-19  🞎 قبل الإصابة بعدوى كوفيد-19  🞎 شعرت بها للمرة الاولى بعد الخروج من المستشفى | | **متى عانيت من حساسية على الجلد ؟** |
| **If you have experienced skin rash before COVID-19 infection, how it is now?** | 🞎 Same as before  🞎 Worse than before  🞎 Better than before  🞎 Completely resolved | |
| 🞎 كما كانت من قبل  🞎 أسوأ من قبل  🞎 أفضل من قبل  🞎 اختفت تماما | | **اذا عانيت من حساسية على الجلد , كيف تصفها الان؟** |

| 1. **Conjunctivitis:** | | |
| --- | --- | --- |
| **التهاب الملتحمة (باطن جفن العين):** | | |
| **Have you experienced conjunctivitis after being discharge from the hospital?** | 🞎 Yes 🞎 No | |
| 🞎 نعم 🞎 لا | | **هل عانيت من التهاب الملتحمة (باطن جفن العين) بعد خروجك من المستشفى؟** |
| **For how long have you experienced conjunctivitis?** | 🞎 4 weeks or less from the onset of COVID-19  🞎 4-12 weeks after the onset of COVID-19  🞎 More than 12 weeks after the onset of COVID-19 | |
| 🞎 4 اسابيع او اقل منذ بداية العدوى  🞎 4-12 اسبوع منذ بداية العدوى  🞎 أكثر من 12 اسبوع منذ بداية العدوى | | **لكم من الوقت عانيت من التهاب الملتحمة (باطن جفن العين)؟** |
| **When was the conjunctivitis experienced?** | 🞎 During COVID-19 infection  🞎 Before COVID-19 infection  🞎 Experienced for the first time after discharge | |
| 🞎 خلال فترة الإصابة بعدوى كوفيد-19  🞎 قبل الإصابة بعدوى كوفيد-19  🞎 شعرت بها للمرة الاولى بعد الخروج من المستشفى | | **متى عانيت من التهاب الملتحمة (باطن جفن العين) ؟** |
| **If you have experienced conjunctivitis before COVID-19 infection, how it is now?** | 🞎 Same as before  🞎 Worse than before  🞎 Better than before  🞎 Completely resolved | |
| 🞎 كما كانت من قبل  🞎 أسوأ من قبل  🞎 أفضل من قبل  🞎 اختفت تماما | | **اذا عانيت من التهاب الملتحمة (باطن جفن العين) , كيف تصفها الان؟** |

Other symptoms? (Same as before/ worse than before/ better than before)?

أعراض اخرى؟ (كما كانت من قبل/ أسوأ من قبل/ أفضل من قبل)

**EQ-5D-5L Quality of life scale:**

| **Mobility:** | 🞎 I have no problems in walking around  🞎 I have slight problems in walking around  🞎 I have moderate problems in walking around  🞎 I have severe problems in walking around  🞎 I am unable to walk around | |
| --- | --- | --- |
| 🞎 ليس لدي اي مشاكل في الحركة والتنقل  🞎 لدي مشاكل طفيفة في الحركة والتنقل  🞎 لدي مشاكل متوسطة في الحركة والتنقل  🞎 لدي مشاكل شديدة في الحركة والتنقل  🞎 لا استطيع الحركة والتنقل على الاطلاق | | **الحركة:** |
| **Self-care:** | 🞎 I have no problems washing or dressing myself  🞎 I have slight problems washing or dressing myself  🞎 I have moderate problems washing or dressing myself  🞎 I have severe problems washing or dressing myself  🞎 I am unable to wash or dress myself | |
| 🞎 ليس لدي اي مشاكل في الاغتسال او ارتداء الملابس  🞎 لدي مشاكل طفيفة في الاغتسال او ارتداء الملابس  🞎 لدي مشاكل متوسطة في الاغتسال او ارتداء الملابس  🞎 لدي مشاكل شديدة في الاغتسال او ارتداء الملابس  🞎 لا استطيع الاغتسال او ارتداء الملابس | | **العناية بالنفس:** |
| **Usual activities (e.g. work, study, housework, family or leisure activities):** | 🞎 I have no problems doing my usual activities  🞎 I have slight problems doing my usual activities  🞎 I have moderate problems doing my usual activities  🞎 I have severe problems doing my usual activities  🞎 I am unable to do my usual activities | |
| 🞎 ليس لدي اي مشاكل في القيام بنشاطاتي الاعتياديّة  🞎 لدي مشاكل طفيفة في القيام بنشاطاتي الاعتياديّة  🞎 لدي مشاكل متوسطة في القيام بنشاطاتي الاعتياديّة  🞎 لدي مشاكل شديدة في القيام بنشاطاتي الاعتياديّة  🞎 لا استطيع القيام بنشاطاتي الاعتياديّة | | **النشاطات الاعتياديّة (العمل, الدراسة, اعمال المنزل, نشاطات عائليّة)** |
| **Pain / discomfort** | 🞎 I have no pain or discomfort  🞎 I have slight pain or discomfort  🞎 I have moderate pain or discomfort  🞎 I have severe pain or discomfort  🞎 I have extreme pain or discomfort | |
| 🞎 ليس لدي اي الم او شعور بعدم الراحة  🞎 اعاني بشكل طفيف من الم او شعور بعدم الراحة  🞎 اعاني بشكل متوسط من الم او شعور بعدم الراحة  🞎 اعاني بشكل شديد من الم او شعور بعدم الراحة  🞎 اعاني بشكل شديد جدا من الم او شعور بعدم الراحة | | **الم / شعور بعدم الراحة؟** |
| **Anxiety / depression** | 🞎 I am not anxious or depressed  🞎 I am slightly anxious or depressed  🞎 I am moderately anxious or depressed  🞎 I am severely anxious or depressed  🞎 I am extremely anxious or depressed | |
| 🞎 ليس لدي اي قلق او اكتئاب  🞎 اعاني بشكل طفيف من القلق او الاكتئاب  🞎 اعاني بشكل متوسط من القلق او الاكتئاب  🞎 اعاني بشكل شديد من القلق او الاكتئاب  🞎 اعاني بشكل شديد جدا من القلق او الاكتئاب | | **الم / شعور بعدم الراحة؟** |
