# Supplementary material for: Reported long-term effects of COVID-19 patients after hospital discharge in Jordan
Source: Medicine (Baltimore). 2023 Sep 22;102(38):e34633. doi: 10.1097/MD.0000000000034633 (PMC10519471; doi:10.1097/MD.0000000000034633)
Supplement: Supplementary file 2 [file medi-102-e34633-s002.docx]

| **Supplementary table 1. Symptoms for less than 1 year follow-up duration** | | | | | | | | |
| --- | --- | --- | --- | --- | --- | --- | --- | --- |
|  |  | **Severity of Illness** | | | **OR (95% CI)** | | | |
| **Symptoms** | **Total (%)**  **n=341** | **Moderate (%)**  **n=33** | **Severe (%)**  **n=106** | **Critical (%)**  **n=202** | | **Severe VS Moderate** | | **Critical VS Moderate** |
| Any of the following symptoms | 314/341 (92.1) | 30/33 (90.9) | 98/106 (92.5) | 168/202 (92.1) | 1.23 (0.31-4.91) | | 1.16 (0.32-4.23) | |
| Extreme fatigue | 213/335 (63.6) | 20/31 (64.5) | 72/105 (68.6) | 121/199 (60.8) | 1.20 (0.52-2.79) | | 0.85 (0.39-1.88) | |
| Cough | 146/333 (43.8) | 15/33 (45.5) | 50/105 (47.6) | 81/195 (41.5) | 1.09 (0.50-2.39) | | 0.85 (0.41-1.79) | |
| Hemoptysis | 10/341 (2.9) | 2/33 (6.1) | 1/106 (0.9) | 7/202 (3.5) | 0.15 (0.01-1.68) | | 0.56 (0.11-2.80) | |
| Sputum production | 68/334 (20.4) | 11/33 (33.4) | 24/105 (22.9) | 33/196 (16.8) | 0.59 (0.25-1.39) | | **0.40 (0.18-0.91)** | |
| Fever | 11/341 (3.2) | 2/33 (6.1) | 4/106 (3.8) | 5/202 (2.5) | 0.61 (0.11-3.48) | | 0.39 (0.07-2.12) | |
| Chills | 47/341 (13.8) | 7/33 (21.2) | 17/106 (16.0) | 23/202 (11.4) | 0.71 (0.27-1.90) | | 0.48 (0.19-1.22) | |
| Nasal congestion | 44/338 (13.0) | 5/33 (15.2) | 14/106 (13.2) | 25/199 (12.6) | 0.85 (0.28-2.57) | | 0.80 (0.28-2.28) | |
| Nosebleed | 10/339 (3.0) | 2/32 (6.3) | 4/106 (3.8) | 4/201 (2.0) | 0.59 (0.10-3.37) | | 0.30 (0.05-1.74) | |
| Dyspnea | 184/333 (55.3) | 21/33 (63.6) | 50/104 (48.1) | 113/196 (57.7) | 0.53 (0.24-1.19) | | 0.78 (0.36-1.67) | |
| Sore Throat | 41/335 (12.2) | 4/33 (12.1) | 10/104 (9.6) | 27/198 (13.6) | 0.77 (0.22-2.64) | | 1.14 (0.37-3.51) | |
| Chest pain | 77/333 (23.1) | 7/32 (21.9) | 27/103 (26.2) | 43/198 (21.7) | 1.27 (0.49-3.27) | | 0.99 (0.40-2.45) | |
| Palpitation | 50/113 (16.0) | 6/32 (18.8) | 18/99 (18.2) | 26/182 (14.3) | 0.96 (0.35-2.68) | | 0.72 (0.27-1.92) | |
| Changes mood Anxiety/Depression | 116/309 (37.5) | 12/30 (40.0) | 31/96 (32.3) | 73/183 (39.9) | 0.72 (0.31-1.67) | | 1.00 (0.45-2.19) | |
| Headache | 51/329 (15.5) | 4/29 (13.8) | 15/105(14.3) | 32/195 (16.4) | 1.04 (0.32-3.41) | | 1.23 (0.40-3.77) | |
| Seizures | 1/341 (0.3) | 0/33 (0.0) | 1/106 (0.9) | 0/202 (0.0) | - | | - | |
| Loss of taste | 57/341 (16.7) | 8/33 (24.2) | 16/106 (15.1) | 33/202 (16.3) | 0.56 (0.21-1.44) | | 0.61 (0.25-1.47) | |
| Loss of smell | 57/340 (16.8) | 8/33 (24.2) | 16/105 (15.2) | 33/202 (16.3) | 0.56 (0.22-1.46) | | 0.61 (0.25-1.47) | |
| Diarrhea | 35/336 (10.4) | 3/33 (9.1) | 13/103 (12.6) | 19/200 (9.5) | 1.44 (0.39-5.42) | | 1.05 (0.29-3.77) | |
| Vomiting | 6/339 (1.8) | 2/33 (6.1) | 1/104 (1.0) | 3/202 (1.5) | 0.15 (0.01-1.72) | | 0.23 (0.04-1.45) | |
| Abdominal pain | 18/337 (5.3) | 5/31 (16.1) | 4/104 (3.9) | 9/202 (4.5) | **0.21 (0.05-0.83)** | | **0.24 (0.08-0.78)** | |
| Myalgia | 118/268 (44.0) | 9/23 (39.1) | 36/83 (43.4) | 73/162 (45.1) | 1.19 (0.46-3.06) | | 1.28 (0.52-3.12) | |
| Skin rash | 23/334 (6.9) | 1/30 (3.3) | 10/106 (9.4) | 12/198 (6.1) | 3.02 (0.37-24.6) | | 1.87 (0.23-14.93) | |
| Conjunctivitis | 6/338 (1.8) | 1/31 (3.2) | 2/106 (1.9) | 3/201 (1.5) | 0.58 (0.05-6.58) | | 0.45(0.05 -4.51) | |
| **mMRC score** |  |  |  |  |  | |  | |
| ≥1 | 67 (48.6) | 10 (62.5) | 15 (40.5) | 42 (49.4) | 0.41 (0.12-1.37) | | 0.59(0.19-1.76) | |
