# Supplementary material for: Reported long-term effects of COVID-19 patients after hospital discharge in Jordan
Source: Medicine (Baltimore). 2023 Sep 22;102(38):e34633. doi: 10.1097/MD.0000000000034633 (PMC10519471; doi:10.1097/MD.0000000000034633)
Supplement: Supplementary file 3 [file medi-102-e34633-s003.docx]

| **Supplementary table 2. Symptoms for more than 1 year follow-up duration** | | | | | | | |
| --- | --- | --- | --- | --- | --- | --- | --- |
|  |  | **Severity of Illness** | | | **OR (95% CI)** | | |
| **Symptoms** | **Total (%)**  **n=353** | **Moderate (%)**  **n=89** | **Severe (%)**  **n=144** | **Critical (%)**  **n=120** | **Severe VS Moderate** | | **Critical VS Moderate** |
| Any of the following symptoms | 335/353 (94.9) | 83/89 (93.3) | 134/144 (93.1) | 118/120 (98.3) | 0.97 (0.34-2.76) | 4.27 (0.84-21.65) | |
| Extreme fatigue | 290/350 (82.9) | 71/89 (79.9) | 115/142 (81.0) | 104/119 (87.4) | 1.08 (0.55-2.10) | 1.76 (0.83-3.72) | |
| Cough | 207/351 (59.0) | 43/88 (48.9) | 92/143 (64.3) | 72/120 (60.0) | **1.89 (1.10-3.24)** | 1.57 (0.90-2.73) | |
| Hemoptysis | 14/353 (4.0) | 3/89 (3.4) | 6/144 (4.2) | 5/120 (4.2) | 1.25 (0.30-5.11) | 1.25 (0.29-5.36) | |
| Sputum production | 89/352 (25.3) | 24/89 (27.0) | 36/143 (25.2) | 29/120 (24.2) | 0.91 (0.50-1.66) | 0.86 (0.46-1.62) | |
| Low grade fever | 4/353 (1.1) | 0/89 (0.0) | 3/144 (2.1) | 1/120 (0.8) | 2.53 (0.26-24.66) | NA | |
| High grade fever | 172/353 (48.7) | 35/89 (39.3) | 75/144 (52.1) | 62/120 (51.7) | 1.68 (0.98-2.87) | 1.65 (0.95-2.88) | |
| Chills | 169/353 (47.9) | 42/89 (47.2) | 71/144 (49.3) | 56/120 (46.7) | 1.09 (0.64-1.85) | 0.98 (0.57-1.70) | |
| Nasal congestion | 76/352 (21.6) | 20/89 (22.5) | 34/143 (23.8) | 22/120 (18.3) | 1.08 (0.57-2.02) | 0.77 (0.39-1.52) | |
| Nosebleed | 10/353 (2.8) | 4/89 (4.5) | 5/144 (3.5) | 1/120 (0.8) | 0.76 (0.20-2.93) | 0.18 (0.02-1.63) | |
| Dyspnea | 239/348 (55.3) | 49/87 (56.3) | 99/141 (70.2) | 91/120 (75.8) | **1.83 (1.05-3.19)** | **2.43 (1.34-4.41)** | |
| Sore Throat | 89/335 (25.2) | 17/89 (19.1) | 30/144 (20.8) | 42/120 (35.0) | 1.11 (0.57-2.17) | **2.28 (1.19-4.36)** | |
| Chest pain | 150/351 (42.7) | 40/88 (45.5) | 51/143 (35.7) | 59/120 (49.2) | 0.67 (0.39-1.14) | 1.16 (0.67-2.01) | |
| Palpitation | 91/344 (26.5) | 16/85 (18.8) | 32/142 (22.5) | 43/117 (36.8) | 1.25 (0.64-2.46) | **2.51 (1.29-4.85)** | |
| Changes mood Anxiety/Depression | 165/343 (48.1) | 41/85 (48.2) | 72/139 (51.8) | 52/119 (43.7) | 1.15 (0.67-1.98) | 0.83 (0.48-1.46) | |
| Headache | 131/346 (37.9) | 32/87 (36.8) | 57/140 (40.7) | 42/119 (35.3) | 1.18 (0.68-2.05) | 0.94 (0.53-1.67) | |
| Seizures | 0/353 (0.0) | 0/89 (0.0) | 0/144 (0.0) | 0/120 (0.0) | NA | NA | |
| Loss of taste | 141/353 (39.9) | 42/89 (47.2) | 55/144 (38.2) | 44/120 (36.7) | 0.69 (0.41-1.18) | 0.65 (0.37-1.13) | |
| Loss of smell | 142/351 (16.8) | 42/88 (47.7) | 57/144 (39.6) | 43/119 (36.1) | 0.72 (0.42-1.23) | 0.62 (0.35-1.09) | |
| Diarrhea | 88/353 (24.9) | 20/89 (22.5) | 40/144 (27.8) | 28/120 (23.3) | 1.33 (0.72-2.46) | 1.05 (0.55-2.02) | |
| Vomiting | 45/352 (12.8) | 10/88 (11.36) | 20/144 (13.9) | 15/120 (12.5) | 1.26 (0.56-2.83) | 1.11 (0.48-2.61) | |
| Abdominal pain | 57/353 (16.2) | 17/89 (19.1) | 23/144 (16.0) | 17/120 (14.2) | 0.81 (0.40-1.61) | 0.70 (0.33-1.46) | |
| Myalgia | 215/317 (67.8) | 48/79 (60.8) | 91/128 (71.1) | 76/110 (69.1) | 1.59 (0.88-2.87) | 1.44 (0.79-2.65) | |
| Skin rash | 20/348 (5.8) | 6/85 (7.1) | 5/144 (3.5) | 9/119 (7.6) | 0.47 (0.14-1.60) | 1.08 (0.37-3.15) | |
| Conjunctivitis | 15/353 (4.25) | 4/89 (4.5) | 7/144 (4.9) | 4/120 (3.3) | 1.09 (0.31-3.82) | 0.73 (0.18 -3.01) | |
| **mMRC score** |  |  |  |  |  |  | |
| ≥1 | 106/190 (55.8) | 19/41 (46.3) | 50/80 (62.5) | 37/69 (53.6) | 1.93 (0.90- 4.14) | 1.33(0.62- 2.91) | |
